# Patient engagement in a national research network: barriers, facilitators, and impacts

**DOI:** 10.1186/s40900-023-00418-5

**Published:** 2023-03-08

**Authors:** Miriam Gonzalez, Tatiana Ogourtsova, Alix Zerbo, Corinne Lalonde, Amy Spurway, Frank Gavin, Keiko Shikako, Jonathan A. Weiss, Annette Majnemer

**Affiliations:** 1 Faculty of Medicine and Health Sciences, School of Physical and Occupational Therapy, McGill University, 3654 Promenade Sir William Osler, Montréal, H3G 1Y5 Canada; 2 Research Institute of the McGill University Health Centre, 1001 Decarie Blvd, Montréal, H4A 3J1 Canada; 3 The Research Center of the Jewish Rehabilitation Hospital, Centre Intégré de Santé Et de Services Sociaux de Laval, Site of Centre for Interdisciplinary Research in Rehabilitation of Greater Montreal, 3205 PI. Alton-Goldbloom, Laval, QC H7V 1R2 Canada; 4 The CHILD-BRIGHT Patient-Oriented Research Network, 5252 Boul de Maisonneuve O., Montréal, H4A 3S5 Canada; 5 Centre for Interdisciplinary Research in Rehabilitation of Greater Montreal (Mackay Site), 7000 Sherbrooke St. West, H4B 1R3 Montréal, Canada; 6 Department of Psychology, Faculty of Health, York University, 4700 Keele St., Toronto, ON M3J 1P3 Canada

**Keywords:** Patient engagement, Research networks, Large teams, Barriers, Facilitators, Impacts, Patient-partners, Researchers

## Abstract

**Background:**

Little is known about patient engagement in the context of large teams or networks. Quantitative data from a larger sample of CHILD-BRIGHT Network members suggest that patient engagement was beneficial and meaningful. To extend our understanding of the barriers, facilitators, and impacts identified by patient-partners and researchers, we conducted this qualitative study.

**Methods:**

Participants completed semi-structured interviews and were recruited from the CHILD-BRIGHT Research Network. A patient-oriented research (POR) approach informed by the SPOR Framework guided the study. The Guidance for Reporting Involvement of Patients and the Public (GRIPP2-SF) was used to report on involvement of patient-partners. The data were analyzed using a qualitative, content analysis approach.

**Results:**

Twenty-five CHILD-BRIGHT Network members (48% patient-partners, 52% researchers) were interviewed on their engagement experiences in the Network’s research projects and in network-wide activities. At the research project level, patient-partners and researchers reported similar barriers and facilitators to engagement. *Barriers* included communication challenges, factors specific to patient-partners, difficulty maintaining engagement over time, and difficulty achieving genuine collaboration. *Facilitators* included communication (e.g., open communication), factors specific to patient-partners (e.g., motivation), and factors such as respect and trust. At the Network level, patient-partners and researchers indicated that time constraints and asking too much of patient-partners were *barriers* to engagement. Both patient-partners and researchers indicated that communication (e.g., regular contacts) *facilitated* their engagement in the Network. Patient-partners also reported that researchers’ characteristics (e.g., openness to feedback) and having a role within the Network facilitated their engagement. Researchers related that providing a variety of activities and establishing meaningful collaborations served as facilitators. In terms of impacts, study participants indicated that POR allowed for: (1) projects to be better aligned with patient-partners’ priorities, (2) collaboration among researchers, patient-partners and families, (3) knowledge translation informed by patient-partner input, and (4) learning opportunities.

**Conclusion:**

Our findings provide evidence of the positive impacts of patient engagement and highlight factors that are important to consider in supporting engagement in large research teams or networks. Based on these findings and in collaboration with patient-partners, we have identified strategies for enhancing authentic engagement of patient-partners in these contexts.

**Supplementary Information:**

The online version contains supplementary material available at 10.1186/s40900-023-00418-5.

## Abstract (summary)

This qualitative research paper seeks to understand patient engagement in large teams and networks. Patient engagement is the meaningful and active partnership of patients on a research team. We aim to understand the factors needed in a research environment that consider and include patients. Patient engagement was measured through interviews with 25 CHILD-BRIGHT Network members, either patient-partners or researchers, about their experiences. In this study, patient-partners were the parents of youth affected by brain-based disorders. We identified factors that made it easier or more difficult for patient-partners to engage with the projects and the network. Additionally, we looked at the impacts of patient engagement as observed by the interviewees. We found that at the project level and network level, the factors that helped engagement and made it difficult to engage were similar for both patient-partners and researchers. At the project level for example, open communication and factors specific to patient-partners (e.g., motivation to contribute) were identified by patient-partners and researchers as helping engagement. Maintaining long-term engagement and ensuring meaningful collaboration were identified as factors that make engagement difficult. At the network level, both patient-partners and researchers noted that communication (e.g., regular follow-cup) made it easier to engage while time constraints and asking too much from patient-partners made engagement more difficult. Finally, interviewees shared that patient engagement impacted patient-partners, researchers, and the research being conducted. Patient engagement helped ensure that the research reflected patient-partners’ priorities, allowed collaboration, and provided patient-partners and researchers with learning opportunities. The results of our research have allowed us to identify strategies that can be used to create more meaningful engagement within large research teams.

**Supplementary Information:**

The online version contains supplementary material available at 10.1186/s40900-023-00418-5.

## Introduction

Canada’s Strategy for Patient-Oriented Research (SPOR) calls for the meaningful engagement of patients as partners throughout the research process and for active collaboration of interdisciplinary teams with patient-partners [1]. Although the terms patients, caregivers, consumers, and citizens are used interchangeably in the literature to refer to patients [2], the Canadian Institutes of Health Research defines the term ‘patient’ within the context of health care as an individual with personal experience of a health issue or condition as well as their informal caregivers [1]. While various definitions of patient engagement have been proposed [2–4], the Canadian Institutes of Health Research defines patient engagement as “meaningful and active collaboration in governance, priority setting, conducting research and knowledge translation" [1]. Patient engagement in research aims to: (1) enhance research relevance, (2) improve patient health outcomes and healthcare delivery, and (3) facilitate uptake of results by intended audiences [1, 5, 6]. Similar international initiatives that promote patient engagement exist in the United States and the United Kingdom [7, 8].

To date, Canada’s SPOR has funded seven national research networks [9]. These research networks: (1) are pan-Canadian communities involving patients, researchers, health care professionals, and other stakeholders, (2) focus on health areas of importance across Canadian provinces and territories, (3) address priorities identified by patients and fund related research projects, (4) oversee Network activities such as providing training, engaging new citizens, measuring impact of patient engagement and, (5) aim to accelerate the translation of research findings into practice [9]. One such network is the Child Health Initiatives Limiting Disability- Brain Research Improving Growth and Health Trajectories (CHILD-BRIGHT) Network (www.child-bright.ca). CHILD-BRIGHT is composed of patient-partners, clinicians, scientists, policymakers, and trainees committed to enhancing the health and well-being of children with brain-based developmental disabilities and their families, with youth with disabilities and parents/caregivers (i.e. ‘patients’) meaningfully engaging in the research. Given Network members (n = 374) come from acrossthe Canadian provinces, virtual modes of connecting (e.g., tele or video conference) are primarily used.

Although research on patient engagement has grown in the last decade [10], few studies have measured patient engagement in childhood disability research where the patient-partner is typically the parent [11]. Further, little is known about patient engagement in the context of large teams or networks. Most of the literature that exists comes from research conducted in the U.S. and is descriptive in nature, highlighting reflections on challenges, successes, and methods used to evaluate engagement [12–19]. One study in the U.S. systematically evaluated the engagement experiences of patient-partners in a patient-oriented research network and identified facilitators of engagement such as communicating clear expectations and assessing engagement on a regular basis [20]. Another study conducted in the context of a Canadian research network examined researchers’ perspectives regarding engagement (e.g., meaning, values, challenges) to inform the implementation of a patient engagement strategy within the network [21]. Key concerns identified by researchers in this study were a lack of evidence on impacts of engagement and the need for education on patient engagement within the research community. Within the CHILD-BRIGHT Network, a pilot patient engagement study was conducted to evaluate the engagement processes of one of the thirteen research projects funded by CHILD-BRIGHT [22]. Overall, the patient engagement experience was highly valued and seen as beneficial, both by parent-partners and by researchers. Project challenges (e.g., feedback logistics), facilitators of engagement (e.g., communication), and engagement impacts (e,g., improved project’s relevance) were noted within the process of patient-engagement. To our knowledge, no Canadian data exist on the engagement experiences (e.g., barriers, facilitators, impacts) of patient-partners and researchers working in a nation-wide research network where members primarily connect through virtual means.

Given the dearth of Canadian research on patient engagement in large research networks and high-quality studies measuring engagement in childhood disability research, it is important to extend our understanding of engagement in these contexts. To evaluate our patient engagement practices at CHILD-BRIGHT and inform strategies to enhance authentic involvement of patient-partners, we first measured engagement using standardized quantitative self-report measures. Preliminary findings suggest that both patient-partners and researchers are satisfied with their level of engagement in the Network and believe that patient engagement meaningfully influences the research being conducted (see Additional file 1). To gain an in-depth understanding of the barriers, facilitators, and impacts of patient engagement identified by patient-partners and researchers, we conducted the current qualitative study.

## Methods

### Framework and design

We used a patient-oriented research approach that involved engaging patient-partners in each phase of this project. Canada’s Strategy for Patient Oriented Research Framework was used as a roadmap for active collaboration [1]. An exploratory descriptive approach was utilized to examine the data collected through open-ended interviews.

### Involving stakeholders in this study

Given the patient-oriented research mandate at the CHILD-BRIGHT Network, two parents of children with brain-based developmental disabilities were research partners on the project. They were recruited through the Network and both had previous experience partnering with academics on research projects. One of the parent-partners received training on interviewing through this project and conducted half of the interviews. Both patient-partners were involved in all phases of the project: from study conceptualization to recruitment and data collection and co-authoring this manuscript and a related research brief (see Additional file 2). One of our patient-partners was a member of CHILD-BRIGHT’s Citizen Engagement Council (CEC) at the time of the study while the other patient partner was the Director of the CEC and member of CHILD-BRIGHT’s Executive and Network Steering Committees). Thus, both patient-partners were highly involved in Network activities such as being on committees as part of our Network’s governance, participating in Network-wide surveys, contributing to newsletters and blogs, acting as patient-partner peer-reviewer of research proposals, and attending conferences. We used the Guidance for Reporting Involvement of Patients and the Public, GRIPP2-SF [23] to report on the involvement of our patient-partners in this study (see Additional file 3).

### Recruitment and study sample

After completing self-report measures of patient engagement [24], CHILD-BRIGHT members (patient-partners [n = 58], researchers [n = 153]), were invited to participate in a half-hour interview through an email request sent by research coordinators (to reach patient-partners and researchers working on any of our 13 projects) and program coordinators (to reach patient-partners and researchers on any of our 7 committees). The opportunity to participate in these interviews was also advertised through Connections, CHILD-BRIGHT’s newsletter. This was meant to provide our members with an opportunity to further enrich our understanding of potential enablers and barriers to patient engagement in a research network. Those who indicated an interest in participating were contacted by phone or email (based on preference) and study information was shared with them.

### Procedure

Ethics approval to conduct the study was received from the McGill University Health Centre’s Research Ethics Board. Interviewers (a Network communications administrator and a patient-partner) received a one-hour orientation from team members regarding the study’s purpose, interview format/techniques, and administrative procedures. Network members who agreed to participate in the interviews were asked to sign a consent form that included information pertaining to elements of informed consent: purpose of the research, research procedures, risks/benefits to participation, voluntary participation, confidentiality, anonymity, and a statement of consent. A field test was conducted during which each interviewer conducted an interview. Interviewers then met with a research team member to discuss problems encountered and to brainstorm solutions to inform a more consistent approach to be used.

Semi-structured, open-ended interviews were conducted between summer and fall of 2020 via Zoom, a video conference platform [25]. Network members were given the option to participate in either English or French. Interviews were conducted at a time that was most convenient to participants and lasted approximately 30 min. In consultation with patient-partners on the team, we decided the interview should last 30 min given interviews were conducted at a time when Network members were facing increased demands on their time (e.g., home schooling their children) due to the COVID-19 pandemic. Elements of informed consent were reviewed prior to each interview. The interview guide (see Additional file 4), specifically developed for this purpose, included questions about: (1) benefits, challenges, and supports to engagement, (2) perceived impacts of engagement, and (3) experience of wider Network engagement. The open-ended questions allowed interviewees to discuss what they considered important. Follow-up questions were used by the interviewers for clarification purposes and to better understand responses provided. Although an honorarium for participation was not offered, interviewees were told that study findings would be shared with Network members, allowing them to gain knowledge in this area.

### Data analysis

All interviews were conducted in English (as per participants’ preference), audio-recorded, transcribed verbatim, and imported into NVivo 11, a qualitative data management software [26]. Descriptive statistics were used to report on participants’ gender and the stakeholder group participants identified with (patient-partner or researcher). A content analysis approach was used to analyze the qualitative data. Labels or codes were first assigned to each segment of content. Codes were then grouped under higher order headings or categories. Categories were then examined to form higher order headings or themes [27]. Two researchers trained in qualitative data analysis coded the data (M.G and T.O). After one researcher coded the first half of the interviews, the two researchers met to discuss coding and the emerging coding scheme. The second researcher then coded the remaining interview data, adding to the existing coding scheme where needed. The data coded by one researcher was reviewed for congruence of coding by the other researcher and discrepancies were resolved through discussion. Additional strategies used to enhance methodological rigour included line-by-line analysis of the transcripts and prolonged engagement with the data. To identify the most common barriers, facilitators, and impacts reported by patient-partners and researchers, number of utterances per barrier, facilitator, and impact were examined for both stakeholder groups.

## Results

A total of 25 CHILD-BRIGHT Network members participated in the interviews: eighteen (72%) were female and seven (32%) were male. Twelve (48%) were patient-partners (all parents) and thirteen (52%) were researchers. One overarching theme and three subthemes emerged through the analysis. The overarching theme was “The engagement experience of CHILD-BRIGHT Network members”. The subthemes were: (1) barriers and facilitators to engagement with research projects, (2) barriers and facilitators to wider Network engagement, and (3) impacts of patient-oriented research. We present the most frequently reported barriers, facilitators, and impacts of engagement and compare the results across stakeholder groups.

### Barriers and facilitators to engagement with research projects

Both patient-partners and researchers reported that barriers to engagement with research projects included: (1) communication challenges, (2) factors specific to patient-partners (e.g., time constraints, lack of related experience), (3) difficulty maintaining engagement over time, and (4) having to learn how to work together to achieve genuine collaboration. Table 1 presents the barriers reported, information about what barriers entailed (e.g., communication challenges, patient-partner factors), and representative quotations. Communication challenges were the most frequently reported barrier by both patient-partners and researchers. These challenges included: having unclear expectations and roles, lack of in-person communication, and being in different time zones. As one patient-partner reported, *“You have parent-advisors from all across Canada. So the time difference made it difficult to coordinate, for all of the people to be able to come on was hard.”* Similarly, a researcher noted, *“I think the weaknesses in this actually is trying to work across so many different time zones.”* Finally, whereas lack of integration of patient-partner feedback into projects was another barrier noted by patient-partners, lack of guidelines about how and when to engage patient-partners was a barrier noted by researchers.

**Table 1:** Barriers to engagement with research projects most frequently reported by CHILD-BRIGHT patient-partners and researchers

Barriers	
Patient-partners (total number of utterances = 57^a^)	Researchers (total number of utterances = 42^b^)
1. Communication challenges (25 utterances) (Unclear expectations and roles; Lack of follow-up; Logistics of meetings; Being in different time zones; Lack of in-person communication; Feeling excluded; Use of academic jargon) *“Sometimes communication can be a challenge in terms of assumptions about what I would think my role would be. They would have a different idea than what I would.”*	1. Communication challenges (11 utterances) (Unclear expectations and roles; Lack of follow-up; Logistics of meetings; Being in different time zones; Lack of in-person communication; Lengthy questionnaires) *“I think closing the loop [follow up on how patient-partner feedback was used] has been a real big challenge for us. Even though we know it's important, and we value it, it's an extra step right that we don't typically do when we're a research project. We just make decisions, and we move forward with them.”*
2. Factors specific to patient-partners (13 utterances) (Time limitation and working schedule; Lack of related experience; Role recognition; Homogeneity of patient-partners*;* Engagement can be too scientific and methodological for patient-partners) ***“****When I started out, I had no idea of how much time [I’d spend]. I'm spending much more time than I expected to spend on it… this varies a lot but certainly in at least two full days a week on average.”*	2. Lack of guidelines, framework, and structure (7 utterances) (Lack of guidelines about: how and when to engage, recruiting patient-partners, infrastructural support, engagement curriculum/framework) *“Very quickly, I realized that these families, we needed a formal curriculum for [patient engagement]. There is a skill set that the rest of us had that these families did not have, as gifted as they were, to do this. So, I feel like there are certain parts of family engagement that require curriculum and training that we did not have.”*
3. Difficulty maintaining engagement over time (6 utterances) *“Waiting, having a patient-partner sit on the sidelines doing nothing for four or five months while [Research Ethics Board] approval is gained, might seem like nothing to the research team, but it may be a whole lot to a particular patient-partner who says, ‘I'm out of the loop completely’.”*	3. Having to learn how to work together and achieve genuine collaboration (5 utterances) *“A big thing that we found early on was that we need to work a little bit to make [the partnership] reciprocal. So that it felt like we weren't always only talking about what we need … and that we were listening to them.”*
4. Having to learn how to work together and achieve genuine collaboration (4 utterances) *“There's been a challenge sometimes in achieving genuine collaboration. Sometimes that means being candid with people. […]. What happens sometimes is there's a dynamic whereby you're there to point out problems and to press for change.”*	4. Difficulty maintaining engagement over time (4 utterances) *“…Sometimes research can be very slow and it’s a bit discouraging sometimes for patient- and family partners to be involved because they’re all excited to be a part of it, but then research projects go on for years and years […], and so I think sometimes that can be a bit disheartening.”*
5. Lack of patient-partner feedback integration (4 utterances) *“Not everything I say is relevant or should be taken into account but I know the researchers on [a project external to CHILD-BRIGHT] are very, very careful, even if they're going to dismiss what we say, … that is not the case with every single researcher that I've come in contact with at CHILD-BRIGHT.”*	5. Factors specific to patient-partners (4 utterances) (Time limitation and working schedule; Lack of related experience; Role recognition; Homogeneity of patient-partners) *“The complexity of their [patient-partners] lives makes it extraordinarily difficult to ask them”*

Utterances reported per barrier or facilitator may not add up to total number of utterances as most frequently reported barriers and facilitators are presented. ^a^refers to the total number of instances patient-partners spoke about barriers to engagement with projects. ^b^refers to the total number of instances researchers spoke about barriers to engagement with projects

Patient-partners and researchers also reported similar facilitators to engagement with research projects: (1) communication (e.g., open communication), (2) factors specific to patient-partners (e.g., motivation), and (3) respect, trust, and reciprocal partnership. Table 2 lists facilitators reported, information about those facilitators (e.g., communication, patient-partner qualities), and sample quotes. The most frequently reported facilitator for both stakeholder groups was communication which included using different methods of communication and holding national or group meetings (see Table 2). For patient-partners, having their feedback integrated into projects, receiving an explanation as to why feedback could not be used, and factors specific to researchers (e.g., openness to collaboration) were also seen as facilitators to engagement. As one patient-partner explained, ***“****There was so much genuine interest from the research team to collaborate, that was why I stayed with it.”* For researchers, flexibility afforded to them with their projects (e.g., extensions, meetings) and in providing patient-partner compensation also facilitated engagement. As one researcher noted, *“We’ve had to extend our timeline a few times. They [CHILD-BRIGHT] have been really supportive throughout the entire process of us doing patient-engaged research.”*

**Table 2:** Facilitators to engagement with research projects most frequently reported by CHILD-BRIGHT patient-partners and researchers

Facilitators	
Patient-partners (total number of utterances = 65^a^)	Researchers (total number of utterances = 69^b^)
1. Communication (29 utterances) (National/group meetings; Using different methods of communication; CHILD-BRIGHT as a safe space to speak up; Open communication; Face-to-face interactions; Check-ins) *“Upon learning that there is a Zoom phone app, one patient-partner shared: “I can [now] walk around with my earbuds and still administer medications or check on my son [during meetings]. So, that was wonderful to know that those resources were available.”*	1. Communication (14 utterances) (Having National/group meetings; Using different methods of communication; Seeing CHILD-BRIGHT as a safe space to speak up; Open communication; Check-ins) *“The way the conferences have been run has been quite a success in terms of bringing a lot of patient family partners together in the conference along with researchers […] I think that’s been a real strength.”*
2. Factors specific to patient-partners (8 utterances) (Experience/skills; Motivation and commitment) *“We all come from different backgrounds. Many of us have several degrees, many of us have our own businesses, or jobs that we do in addition to parenting and that can actually be useful too in the work that [researchers] do.”*	2. Factors specific to patient-partners (6 utterances) (Experience/skills; Motivation and commitment; Bringing different perspectives to the table) *“Sharing perspectives has been [very useful] […] the more perspectives you can bring in from people from different backgrounds that are connected somehow, or stakeholders, I think that can only improve the direction.”*
3. Respect, trust, and partnership (5 utterances) (Importance of mutual respect and trust between pt-partners and researchers; Importance of reciprocal partnerships where both parties benefit) *“When you take the time and effort to do that face to face, and everybody gets to know one another and knows what they're there for, why they're there and what they hope to get out of it, you do have that kind of respect and trust that then informs the rest of the engagement.”*	3. Flexibility (6 utterances) (CHILD-BRIGHT providing flexibility to researchers in participation, extensions, scheduling meetings) ***“****For our project we’ve had to extend our timeline a few times and CHILD-BRIGHT hasn’t put up any barriers in doing that. In fact, they’ve been very supportive knowing that to do work at the level of engagement that they want and that we want, it was a no-brainer to extend the timeline.”*
4. Factors specific to researchers (5 utterances) (Openness of researchers to feedback and collaboration; Previous connection to researchers) *“Sometimes it comes down to really simplistic things. For one project I was with previously, we all got together at a research conference. It was nice when they said, ‘We want you to present the poster’. I thought that was just really wonderful in the sense of saying, ‘Well, you’re a part of the team, you can explain as well.’”*	4. Respect, trust and partnership (5 utterances) (importance of mutual respect and trust between patient-partners and researchers; importance of reciprocal partnerships where both parties benefit) *“Entering into these partnerships with respect, it is very important”*
5. Integrating feedback and explaining why feedback can’t be used (4 utterances) *“They validated what we talked about, they didn’t gloss it over like a professional can do or simplify it […] they [researchers] listen, they also model how to integrate different perspectives.”*	5. Compensation and flexibility in compensation (5 utterances) (Having compensation guidelines; Offering more based on contribution) *“In terms of the compensation process, there's a standard amount of compensation, but if this particular person has done XYZ, we would recommend an additional amount for that person.”*

Utterances reported per barrier or facilitator may not add up to total number of utterances as most frequently reported barriers and facilitators are presented. ^a^refers to the total number of instances patient-partners spoke about facilitators to engagement with projects. ^b^refers to the total number of instances researchers spoke about facilitators to engagement with projects

### Barriers and facilitators to wider network engagement

Researchers spoke about one barrier to Network engagement: the time commitment required to engage patient-partners and asking too much of them (see Table 3). As one researcher explained, *“I feel that would be a barrier to getting any help at all. If the ask was too big.”* While patient-partners also reported that asking too much of them and the time commitment required for engagement were barriers to Network engagement, the most frequently reported barrier by patient-partners was communication. Communication challenges such as lack of information about CHILD-BRIGHT activities and receiving too many emails from the Network were noted by patient-partners: *“I think there’s too many emails. It’s tough when my email fills up.”* Additional barriers reported by patient-partners include power imbalances and factors specific to patient-partners such as feeling uncomfortable speaking up. One patient-partner noted: *“If I wasn’t there, I don’t think anyone [other patient-partners] would’ve said something necessarily. It’s like they’re too scared to ask what’s happening.”*

**Table 3:** Barriers to wider Network engagement most frequently reported by CHILD-BRIGHT patient-partners and researchers

Barriers	
Patient-partners (total number of utterances = 38^a^)	Researchers (total number of utterances = 9^c^)
1. Communication challenges (13 utterances) (Lack of information about CHILD-BRIGHT Network activities/initiatives, Lack of information about research projects; Lack of plain language when communicating; Network communication issue^b^; Too many emails) *“Language is a roadblock. It’s up to people like me to go and remind everybody [to use plain language]."*	1. Time commitment required and asking too much of patient-partners (4 utterances) *“…But to parse our time by all of the little sub-studies that we’re asked to participate in […] There’s a feeling of obligation, there’s a feeling of gratefulness, there’s a feeling of wanting to give back and there an overwhelming feeling of guilt for not being able to keep up with all these things. “*
2. Factors specific to patient-partners (8 utterances) (Limited time; Mismatch between the patient-partner’s experience/interests/views and the research focus; Patient-partners are uncomfortable speaking up) *“A lot of the very pediatric-focused questions that CHILD-BRIGHT is asking, I kind of feel like I'm not necessarily the best person to answer those questions a lot of the time.”*	
3. Time commitment required and asking too much of patient-partners (7 utterances) *“The time commitment, it’s, it’s huge, you know, […] It’s just a huge commitment. I’m considering in the next round, you know, whether I stay or just, I don’t know. I love it, but I have to figure out if I can do it.”*	
4. Power imbalances (6 utterances) (Patient-partners not seen as equal to researchers) *“It is an interesting power dynamic if someone has more power […] and in some ways because the researchers have all the money, they have all the power. […] So, there is that power dynamic, regardless of these labels with experts and things.”*	

Utterances reported per barrier and facilitator may not add up to total number of utterances as most frequently reported barriers and facilitators are presented. ^a^refers to the total number of instances patient-partners spoke about barriers to wider Network engagement with projects. ^b^Network communication issue refers to how to ensure information reaches everyone (executive team, patient-partners, etc.). ^c^refers to the total number of instances researchers spoke about barriers to wider Network engagement

In terms of facilitators, both patient-partners and researchers indicated that communication (e.g., regular follow-ups, different methods of communication) was a facilitator to Network engagement (see Table 4). Whereas communication was the most frequently reported facilitator by researchers, factors specific to researchers (e.g., openness of researchers to feedback, liking the researchers) was the most frequently reported facilitator by patient-partners. Providing a variety of engagement opportunities and establishing meaningful collaborations were also facilitators reported by researchers: “*Some of them [patient-partners], they are not just involved in one thing. Some are on other Network committees or on other projects. So, I think those people feel more connected to the Network.”* Other facilitators reported by patient-partners included factors specific to patient-partners (e.g., motivation to contribute, not being intimidated), having a role within the Network, and having a supportive Network. As one patient-partner noted: *“You know, I feel part of the Network because I have such an integral role.”*

**Table 4:** Facilitators to wider Network engagement most frequently reported by CHILD-BRIGHT patient-partners and researchers

Facilitators	
Patient-partners (total number of utterances = 40^a^)	Researchers (total number of utterances = 15^b^)
1. Factors specific to researchers (14 utterances) (Openness of researchers to feedback; Liking the researchers; Having a previous work relationship with researcher; Sense of commitment to their research) *“I would say that there are some really amazing researchers in CHILD-BRIGHT who are very, very supportive of patient-partners and I think that’s important to say.”*	1. Communication and having a supportive Network (6 utterances) (Regular follow-ups, various communication methods) *“In terms of support, I find the Network is very open to hearing about other ways to engage with patients and families… I think that's something that I really like about the Network, being able to reach out.”*
2. Factors specific to patient-partners (10 utterances) (Motivation to contribute; Not being intimidated or scared) *“The benefit is we're clearing the brush away as we're forging the path and we're right at the front there. I think the intrinsic benefit is giving me that energy to keep doing that.”*	2. Providing different engagement opportunities and establishing meaningful collaborations with patient-partners (6 utterances) *“I mean we’re talking about people who have so many demands on their time, that we try very hard not to make demands that are not, that are not really important. So we don’t trivialize their involvement. We structure it so that they are doing things that make a difference.”*
3. Communication (9 utterances) (Regular follow-ups; Different methods of communication (newsletter, conferences, meeting); Accessible information (accessible format, clear, easy to ask and get questions answered) *“They're very good at, in meetings, making sure that it's in accessible format for everyone and providing translations and everything, so that's good. If we ever have any questions it's always easy to follow up […] it's really easy to reach out… and have all your questions answered so that stuff is great.”*	
4. Having a role within the Network or having a supportive Network (5 utterances) *“I had a very strong relationship with the researchers, and I got the newsletters but I wouldn't sayI understood what the Network was or did prior to becoming [role within Network}”*	

Utterances reported per barrier or facilitator may not add up to total number of utterances as most frequently reported barriers and facilitators are presented. ^a^refers to the total number of instances patient-partners spoke about facilitators to wider Network engagement. ^b^refers to the total number of instances researchers spoke about facilitators to wider Network engagement

### Impacts of patient-oriented research

Table 5 highlights the most frequently reported impacts by stakeholder group with representative quotations. Similar impacts were reported by patient-partners and researchers: (1) projects being better aligned with patient-partner priorities, (2) co-creation and collaboration among researchers, patient-partners, and families, (3) knowledge translation being facilitated by patient-partner input, and (4) learning opportunities. Although learning was reported by both stakeholder groups, researchers spoke about the opportunity to learn about patient-oriented research together whereas patient-partners spoke about research-related learning opportunities such as co-presenting at conferences or learning more about a topic or health condition. As one patient-partner put it: *“Until I got involved with CHILD-BRIGHT, I was unaware of all these varieties, like flowers in a garden that our children with disabilities can be.”* Having the opportunity to meet and connect with other patient-partners and amplifying patient-partners’ voices were additional impacts reported by patient-partners and researchers respectively.

**Table 5:** Impacts of patient-oriented research reported by CHILD-BRIGHT patient-partners and researchers

Impacts of patient-oriented research reported by	
Patient-partners (total number of utterances = 97^a^)	Researchers (total number of utterances = 100^b^)
1. Projects are better aligned with patient-partner priorities and lived experiences (25 utterances) *“This particular project has been really lovely in the way that they have really taken all of our input and our perspectives, because the end user is folks like us.”*	1. Projects are better aligned with patient-partner priorities and lived experiences (32 utterances) *“It ensures that the research is practically oriented and applied, and the language used is simple. There are often family members or self-advocates that are really pushing for the research to be used, and relevant and asking pertinent questions. So I think it does change the tone, for sure.”*
2. Patient-partners are provided with learning opportunities (22 utterances) (e.g., co-present at conferences, attend patient-engagement workshops, learn about others’ circumstances and health conditions) *“And I think sometimes it comes down to really simplistic things* *With one project I was with previously, we all got together at a research conference. It was nice they said, ‘we want you to present the poster’. And I thought that was just really, wonderful in the sense of saying, ‘well you’re a part of the team, you can explain as well.”*	2. Co-creation and collaboration among researchers, patient-partners, and families increases (27 utterances) *“Having the patient-partners very much involved makes any strategic decisions more realistic, more impactful. So, I think for strategic decisions, having patient-partners is really important to make sure that the patient experience and patient expertise is really influencing our decision making.”*
3. Co-creation and collaboration among researchers, patient-partners, and families increases (18 utterances) *“I think that a lot of how I frame things [as a parent-partner], or how I look at things has had a really big impact in how we're reporting, how we're measuring, how we're designing the program…”*	3. Knowledge translation is facilitated by patient-partner input (11 utterances) *“The incredible students that we’ve had have been able to listen to the parents’ feedback, take very specific feedback and turn it into resources for families.”*
4. Knowledge translation is facilitated by patient-partner input (10 utterances) *“ We're redesigning a program that was made for a certain cohort of the disabled population and now we're rejigging it so the videos have to change, some of the language has to change […] because it’s for different disabilities, for children or for parents of children with different kind of disability.”*	4. Members can learn about patient-oriented research together (6 utterances) *“I think we try to be open as much as possible and did see it as a learning process together and I think that was appreciated that we were open in that regards.”*
5. Members can meet others, share experiences, increase knowledge and know they are not alone (8 utterances) *“I would count several of the parent advisors as friends now, or certainly colleagues that I can reach out to on issues not related to the study. So yes, it has enhanced my knowledge of childhood disability… I have been able to interact with parents with kids who have different kinds of disabilities or comorbidities and also different challenges…. I’ve had the chance to meet with some parents as well, which I’m very happy about.”*	5. The voices of patient-partners are amplified (5 utterances) *“By having these parents who are willing—they're very busy but, they're willing because they think it's important to have their voices heard and to speak on behalf of other parents—I think that having that opportunity is really helpful for the study.”*

Utterances reported per barrier or facilitator may not add up to total number of utterances as most frequently reported barriers and facilitators are presented. ^a^refers to the total number of instances patient-partners spoke about impacts of patient-oriented research. ^b^refers to the total number of instances researchers spoke about impacts of patient-oriented research

### Stakeholder involvement

Sample results of engaging patient-partners in this study include: (1) improved readability of study materials (e.g., consent form, interview guide), (2) acquisition of interviewing skills by one of our patient-partners, (3) assistance in promoting interview participation among patient-partners, (4) co-authorship of this manuscript (e.g., provided a critical reflection of study involvement shared in the Discussion section), and (5) development of strategies (based on study findings) to support engagement in large research teams as reported in Table 6.

**Table 6:** Strategies to support engagement in large research teams and networks based on study findings

Factors and related strategies	
*Communication* •Have clearly defined roles, tasks, and expectations for patient-partners at each research phase •Share time commitment information and compensation information from the start •Have regular follow-ups/check-ins re: tasks, project updates •Ask patient-partners if the “ask” is too big. If yes, break task into smaller components and get more than one patient-partner to work on tasks •Encourage in-person meetings (e.g., group meetings) and face-to-face communication •Use different methods of communication (e.g., phone, Zoom, in-person meetings or conferences) •Foster open channels of communication so patient-partners feel comfortable speaking up, asking questions, and accepting/declining to participate in different tasks •Avoid academic jargon •Share with patient-partners when their feedback has been integrated into projects. Share reasons for not integrating their feedback •Share information about Network activities/initiatives •Ensure information shared with patient-partners is in accessible format (e.g., clear, lay language) and there is someone that can be contacted for questions/additional information •Ensure information is available in both English and French	*Training & Support* •Encourage both patient-partners and researchers to participate in training opportunities regarding: Research process and unpredictability of research Patient-oriented research (e.g., guidelines, framework) Working together/Collaboration How to maintain engagement throughout the research process •Offer flexible, learning opportunities (e.g., workshops, online modules, webinars, courses etc.) •Have compensation guidelines •Remain flexible regarding compensation (e.g., offer more based on contribution), project extensions, scheduling meetings •Ensure that compensation does not affect patient-partners’ eligibility for disability support payments etc •Provide different and meaningful engagement opportunities
*Factors specific to patient-partners* •Ensure there is a good match between patient-partner interest/experience and research focus •Remain flexible regarding: How and when patient-partners wish to contribute Time allotted for patient-partners to contribute and provide feedback •Encourage patient-partners who do not have research experience to take up training opportunities •Acknowledge role of patient-partners and contributions made •Acknowledge/Provide encouragement for motivation and commitment	*Factors specific to researchers* •Encourage openness to receive feedback from patient-partners and bring different perspectives to the table •Nurture relationship with patient-partner(s) •Demonstrate commitment to working with patient-partners and research project(s)
*Power Imbalances* •Establish advisory councils/committees that contribute to decision making throughout the research process •Have more than one patient-partner on a given team •Engage in critical reflection of unconscious biases (e.g., privilege as researchers) and identify strategies that can be used to adjust related behaviours	*Relationship-building* •Encourage mutual respect and trust •Encourage reciprocal partnerships (both patient-partners and researchers benefit) •Engage patient-partners throughout the research process, from the very beginning (not as an afterthought or tokenism)

## Discussion

The objective of this study was to gain further in-depth understanding of the barriers, facilitators, and impacts of patient engagement, already identified by patient-partners and researchers through the quantitative assessments of engagement conducted at the CHILD-BRIGHT Network. Given that research on patient-engagement in the context of research networks is in its infancy and that there are few high-quality studies measuring engagement in childhood disability research, this study adds to the knowledge base regarding engagement in research networks and methods for measuring engagement in this context. Our findings provide evidence of the positive impacts of patient engagement (e.g., co-creating with patient-partners, facilitating knowledge translation) and highlight factors (e.g., trust between patient-partners and researchers, open communication) that are critical for supporting engagement in large research teams and networks. Based on these findings and in collaboration with patient-partners, we have identified strategies or best practices for enhancing authentic engagement of patient-partners in these contexts.

### Barriers and facilitators of engagement

Our findings regarding barriers and facilitators to engagement with research projects and wider Network engagement are consistent with the larger body of evidence on barriers and facilitators to engagement with research projects in different health-related applications [6, 10, 11, 20, 22, 28–34]. One interesting finding is that only researchers identified compensation and flexibility in compensation as a facilitator to engagement with research projects. Compensation was not identified as a facilitator nor barrier to engagement by patient-partners. Whether this means that patient-partners at CHILD-BRIGHT are satisfied with the compensation they receive or felt uncomfortable bringing this up requires further investigation.

Some of the barriers and facilitators to wider Network engagement reported in this study have also been identified as barriers and drivers to engagement in a few studies of research networks that focus on other populations [20, 21]. For instance, in Nowell’s [20] evaluation of patient-partners’ engagement in a U.S. patient-powered research network for rheumatologic conditions, communication (e.g., different modes of communication) was identified as a key facilitator to engagement. In Carroll et al. investigation of Canadian researchers’ perspectives regarding challenges to engagement in a cardiovascular research network, barriers to engagement similar to our findings were identified: communication challenges (e.g., lack of role clarity, research jargon), factors specific to patient-partners (e.g., lack of related experience, are uncomfortable speaking up), power imbalances, and the time commitment required [21]. Whereas researchers in this study reported that the time commitment required for patient engagement was a barrier to wider Network engagement, patient-partners reported all four barriers noted above. The finding that researchers in our study identified time commitment as the major barrier to wider Network engagement may reflect researchers’ limited time and resources for research and engagement at the time of our data collection (i.e. a few months into the COVID-19 pandemic) [35]. Researchers’ perspectives on barriers to wider Network engagement need to be further explored.

The limited data on barriers and facilitators to engagement in large research networks has hindered the formulation of guidelines for engaging patient-partners in these contexts. Our findings point to factors that should be considered when devising strategies for this purpose. For instance, barriers identified in this study (e.g., difficulty maintaining engagement over time, lack of engagement guidelines, having to learn how to work together) highlight the need to support and guide both patient-partners and researchers in conducting patient-oriented research. Providing flexible learning opportunities (e.g., information on the research process and/or patient-engagement courses, workshops, or online modules) tailored to the needs of both researchers and patient-partners may prove beneficial in attenuating potential barriers to engagement. To reduce power imbalances, another barrier reported in this study, researchers may wish to use strategies such as establishing advisory councils or engaging in critical reflection of unconscious biases (e.g., beliefs or stereotypes we hold as researchers) and how to address those biases [34, 36].

Our findings also point to other factors that may impact meaningful engagement and require attention. Communication and factors specific to patient-partners were reported as both barriers (e.g., communication challenges such as unclear expectations, patient-partner lack of related experience) and facilitators (e.g., using different methods of communication, patient-partner commitment) to engagement with research projects and wider Network engagement. Patient-partner feedback was also reported as a facilitator (when used by researchers) and a barrier (when not used by researchers) to engagement with projects. Some strategies that may facilitate engagement include communicating clearly about expectations and roles at each phase of the project, remaining flexible regarding patient-partners’ ability to contribute, and providing information to patient-partners as to why their feedback cannot be used. More formal mentorship for parent-partners and for researchers by an experienced parent-partner can also provide more tailored guidance. In collaboration with patient-partners and based on our findings, we further identified additional strategies that can be used to facilitate engagement in large research teams or networks (see Table 6). These strategies pertain to training and support (e.g., remaining flexible regarding compensation, ensuring compensation does not affect patient partners’ eligibility for disability support), factors specific to researchers (e.g., demonstrate commitment to working with patient-partners), communication (e.g., having clearly defines roles, tasks, and expectations), factors specific to patient-partners (e.g., remain flexible s to how and when patient partners wish to contribute), power imbalances (e.g., establish advisory councils that contribute to decision making), and relationship building (e.g., encourage mutual respect and trust).

### Impacts of engagement

Our findings suggest that patient-oriented research can have meaningful impacts, both to the research (e.g., study materials) and to the individuals (e.g., learning interviewing skills) involved. Enhancing project relevance to target populations, co-creating with patient-partners (e.g., study material, research direction), facilitating knowledge translation, and having learning opportunities were reported as potential impacts by both patient-partners and researchers. Our findings are consistent with: (1) the results of a review on the benefits of engagement for researchers and parent co-researchers [11], (2) the findings of a study that examined the benefits of engagement as perceived by researchers in a Canadian research network [21], (3) the larger body of literature on impacts of engagement in the context of research projects [6, 10, 22, 29, 33, 37, 38], and (4) Canadian Institute for Health research priorities for patient engagement of collaboration, co-creation, and facilitating knowledge translation [1]. The finding that the impacts of patient engagement identified in this study (conducted in the context of a large team or network) were similar to the impacts that have been reported in the context of research projects where teams are typically smaller suggests that factors other than team size (e.g., when and how patient-partners are involved) may play a more significant role in determining whether patient engagement is perceived as having impact. The relationship between engagement process variables and perceived impacts needs to be further explored.

Another impact reported by patient-partners was meeting with others to share experiences. The opportunity to meet socially with others going through similar experiences has been previously reported as an impact [38] and suggests that through these opportunities, engagement in research facilitates access to networking support and creation of shared purpose among patient-partners. Finally, although negative impacts of engagement have been previously reported (e.g., increased cost and additional time it takes to engage and collaborate with patient-partners) [10], all impacts reported in this study were positive. This may be a result of the convenience sample who elected to participate in the current study or the lack of explicitly asking about the potential negative impact. Whether similar findings will be obtained in future assessments of engagement at CHILD-BRIGHT, with broader recruitment strategies and when members are probed further about the nuances of impact, awaits further investigation.

### Stakeholder involvement and reflection

The involvement of patient-partners positively influenced various aspects of this study (see results section). This may have been related to: (1) patient-partners’ prior experience collaborating with researchers through their involvement with the CHILD-BRIGHT Network, (2) researchers’ experience involving patient-partners in research, and (3) engaging patient-partners from study inception to completion. We asked one of our patient-partners to reflect on their engagement in this study. The reflection presented below identifies factors that served as facilitators to engagement and that can also serve as barriers when not managed well:

*“Like many patient-partners I found that my interest in contributing to the work of the Network and my ability to do so were intimately connected to the quality of my relationships with researchers, other patient-partners, and administrative staff. Do people use one another's names and which names are used? Do people listen closely to one another? Is there evidence of tokenism? Supposedly little things can make a big difference: how much notice is given of a change in the date or time of a meeting, how far ahead of a meeting the agenda arrives, whether a reimbursement process is complex or easy, drawn-out or prompt, etc. It also matters whether we can see the actual impacts of our contributions on processes and products. We get involved to make a difference.”*

### Strengths and limitations

While previous research conducted in the context of research networks has examined the engagement perspectives of researchers [21], one strength of our study is that both researchers and patient-partners contributed to the study’s data collection and emerging themes. Thus, study findings will inform strategies used by our Network to support future engagement of both stakeholder groups. Another strength is that patient-partners from the CHILD-BRIGHT Network’s Citizen Engagement Council (https://www.child-bright.ca/citizen-engagement-council) reviewed, provided feedback, and validated the findings and strategies to support engagement in large teams or networks. Finally, our sample was considered sufficient as theoretical saturation was achieved. Nonetheless, there are limitations. First, our sample provided data on the experience of patient-partners and researchers affiliated with a specific childhood disability network. Replication of our findings in networks focusing on other populations (e.g., those living with diabetes) or engaging different groups (e.g., seniors) would contribute to the validity of the findings. Second, biases inherent in self-report such as recall bias as well as social desirability bias (e.g., being interviewed by CHILD-BRIGHT members) may have affected the findings. Third, while recruitment emails were circulated across a large network, it is possible that only those with positive experiences or very negative experiences with patient-oriented research chose to participate, with a possibility of sample bias. Fourth, as our Network moves forward with its work, periodic longitudinal assessment of barriers, facilitators, and impacts of engagement using both qualitative and quantitative measures would allow for greater understanding of change over time. Collecting information about study participants beyond gender and stakeholder group (e.g., education, ethnicity, household income) would also allow for nuanced understanding of the findings. Finally, while we identified the importance of communication as a factor that can facilitate or hinder engagement, further work is needed that details how communication is experienced as a barrier or facilitator and what contributes to these experiences.

## Conclusions

With the increase of research on patient engagement, it is important to understand the subtleties of patient-oriented processes (e.g., barriers, facilitators) and impacts of engagement. Our findings provide evidence of several personal and research-related impacts of patient engagement, highlight factors that are important to consider in supporting engagement in the context of large research teams or networks, and can begin to inform best practice guidelines for engaging parents as co-researchers. Based on these findings as well as those from quantitative measures of patient engagement, CHILD-BRIGHT is currently developing a patient-oriented research toolkit for child health researchers which will include tip sheets on patient engagement directed at youth with disabilities (NYAP & SibYAC's 10 Tips for Researchers—CHILD-BRIGHT Network), patient-partners (ENG_Tips for Patient-Partners_Oct 2021 (squarespace.com), and researchers (ENG_Tips for Researchers_Oct 2021 (squarespace.com). The toolkit will also include planning templates and contracts, onboarding materials, and compensation guidelines. The work of our Network’s Training Program, Parent Liaison (mentor), and National Youth Advisory Panel is informed by the results of this study, influencing our future strategic directions in support of authentic engagement by patient-partners in our Network. Given the potential barriers reported by patient-partners and researchers in this study, future research could explore the optimal training needs of both stakeholder groups in patient-oriented research networks. This could contribute to ensuring that in the future, both patient-partners and researchers are well supported when conducting patient-oriented research in those contexts.

## Supplementary Information

**Additional file 1**. “Stakeholder Engagement in CHILD-BRIGHT Network” contains the October 2021 Engagement evaluation report with preliminary findings produced by CHILD-BRIGHT.

**Additional file 2**. “Patient engagement in CHILD-BRIGHT’s patient-oriented research Network: Scratching beneath the surface” contains the April 2022 research brief produced by CHILD-BRIGHT.

**Additional file 3**. “GRIPP2 PPI reporting Checklist” contains the results of the GRIPP2 short form reporting checklist.

**Additional file 4**. “Interview Guide Questions” contains the qualitative interview questions used for patient-partners and researchers.

## Data Availability

The data that support the findings of this study are not publicly available due to privacy and/or ethical restrictions. However, they are available from the CHILD-BRIGHT Network data access committee (admin@childbright.ca) for researchers who meet the criteria for access to confidential data.

## Abbreviations

POR: Patient-oriented research
GRIPP2-SF: Guidance for Reporting Involvement of Patients and the Public
SPOR: Strategy for Patient-Oriented Research
CHILD-BRIGHT: Child Health Initiatives Limiting Disability-Brain Research Improving Growth and Health Trajectories
