# Supplementary material for: Patient engagement in a national research network: barriers, facilitators, and impacts
Source: Res Involv Engagem. 2023 Mar 8;9:7. doi: 10.1186/s40900-023-00418-5 (PMC9993369; doi:10.1186/s40900-023-00418-5)
Supplement: Supplementary file 1 — Additional file 1. “Stakeholder Engagement in CHILD-BRIGHT Network” contains the October 2021 Engagement evaluation report with preliminary findings produced by CHILD-BRIGHT. [file 40900_2023_418_MOESM1_ESM.pdf]

CHILD-BRIGHT NETWORK

OCTOBER 2021

Stakeholder Engagement in the CHILD-BRIGHT Network

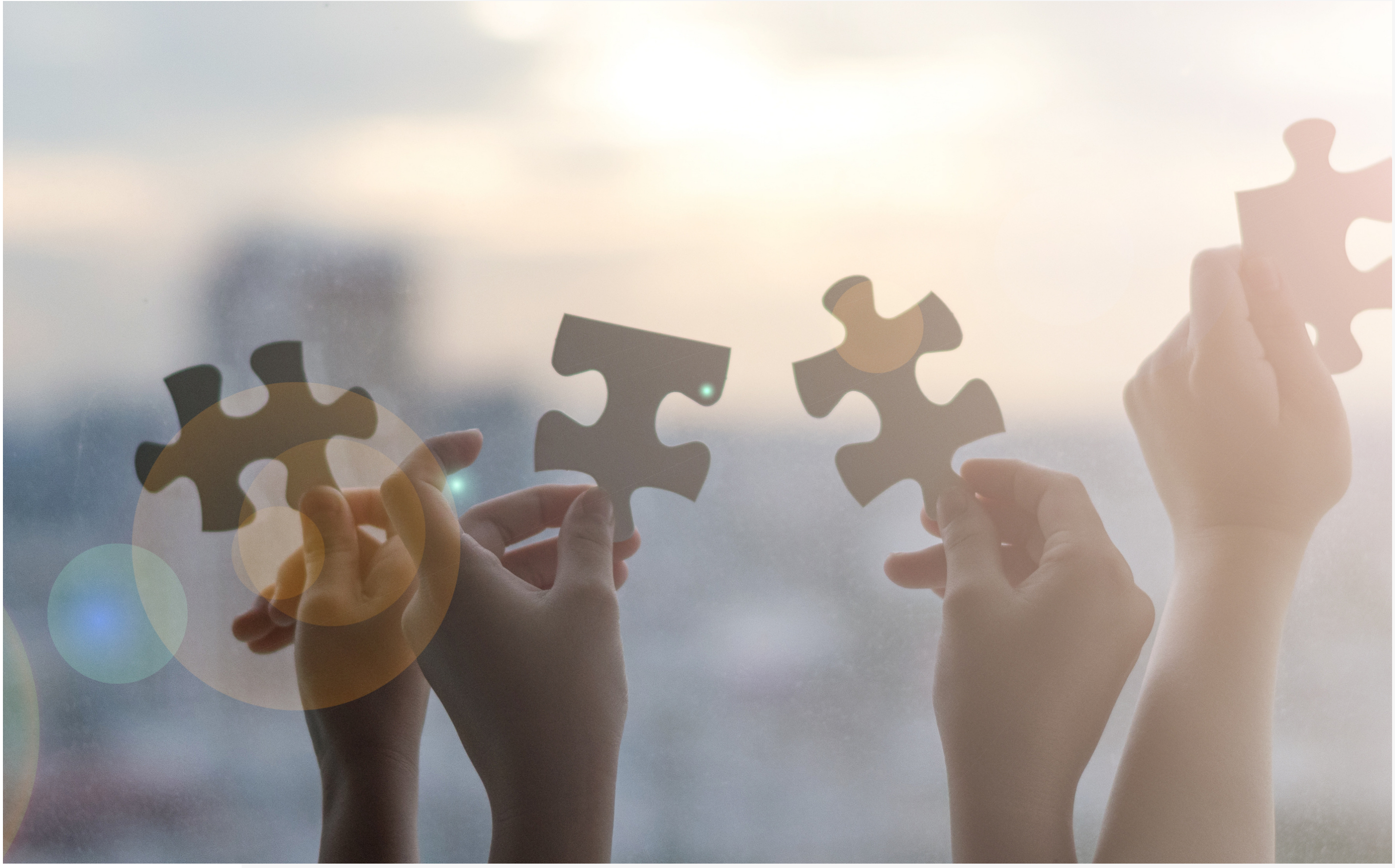

Part 1

Stakeholder engagement in the CHILD-BRIGHT Network, 2018 to 2020

Preliminary findings

CHILD-BRIGHT Network

Strategy for Patient-Oriented Research
SPOR
Putting Patients First

Introduction

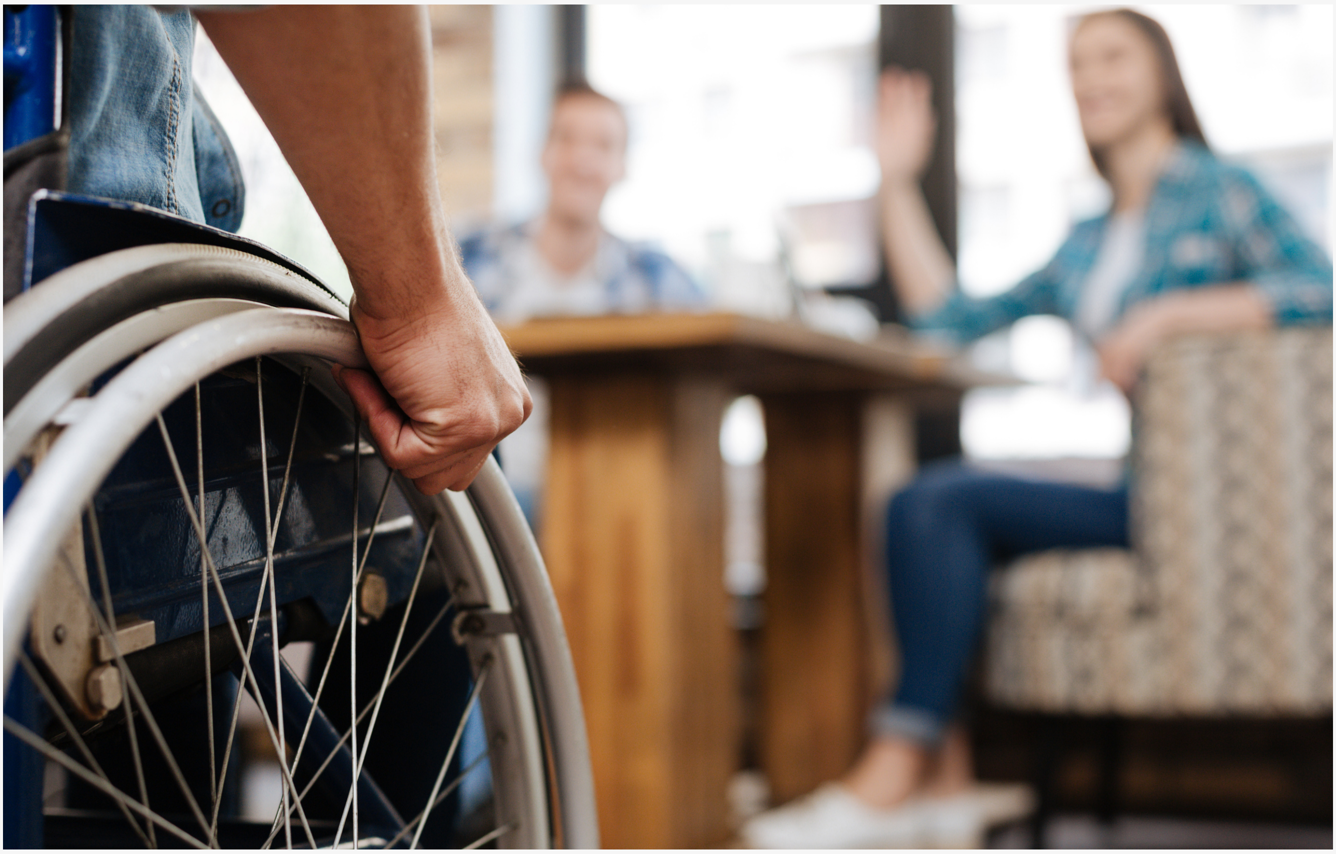

The CHILD-BRIGHT Network is dedicated to patient-oriented research. Various stakeholders including patient-partners (youth, parents), researchers and clinicians, are involved at different levels of the Network and contribute as committee members and/or as research project advisors. The Network is committed to continuously improving its level of engagement and collaboration with patient-partners. Therefore, evaluating the Network's patient engagement and its impacts is an essential ongoing activity.

Patient-oriented research and patient engagement evaluation are rapidly evolving fields. In this brief, which is Part 1 of a three-part series on patient engagement, we are presenting preliminary findings of our patient engagement evaluations and describing our journey as a Network in refining our approaches.

Parts 2 and 3 of this series share the results of our survey about engagement during the COVID-19 pandemic and the findings of a series of interviews with our network members about patient engagement, respectively.

How did it all start?

In **2018, we launched our first engagement evaluation.** We were interested in how different Network members experienced their involvement. We invited researchers, patient-partners (including parents and youth), trainees, and committee members to complete an online survey, the **Community-Based Participatory Research (CBPR)[1] questionnaire.**

What is the CBPR?

The CBPR is an evaluation tool that looks at the degree of different kinds of stakeholder involvement and partnership in the research process, and how researchers are engaging with various stakeholders, including patient-partners. There were two different CBPR versions. One version was sent to various stakeholders who interacted in different ways with research projects. This 'stakeholder version' was sent to patient-partners, staff or trainees, co-investigators, and committee members who informed CHILD-BRIGHT patient-oriented work. It asked respondents about their experiences engaging with CHILD-BRIGHT projects, including project planning, data analysis, and providing input for decision-making, and the level of partnership and trust they felt in the process. The other version of the CBPR was sent to the lead researchers who were involved in CHILD-BRIGHT research projects ('principal investigators'). This version asked the researchers how they felt they engaged with stakeholders and how well they thought stakeholders were engaged in CHILD-BRIGHT.

We sent the CBPR to stakeholders in three consecutive years: 2018, 2019, and 2020.

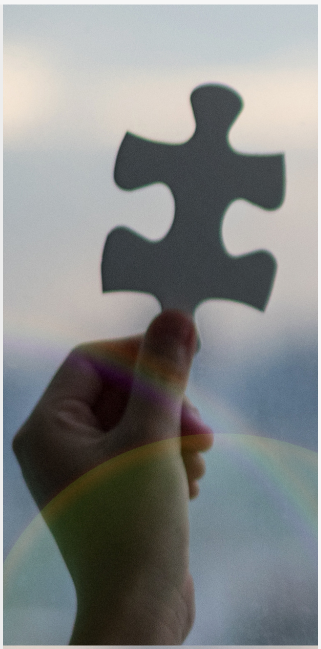

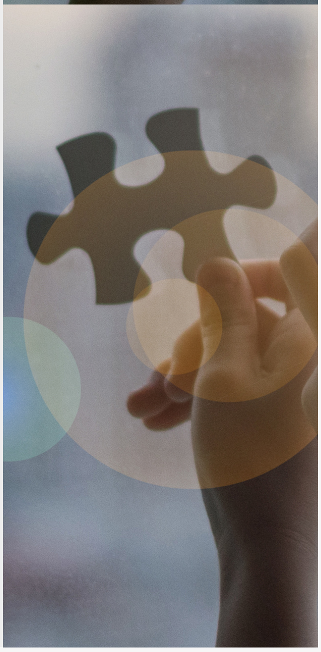

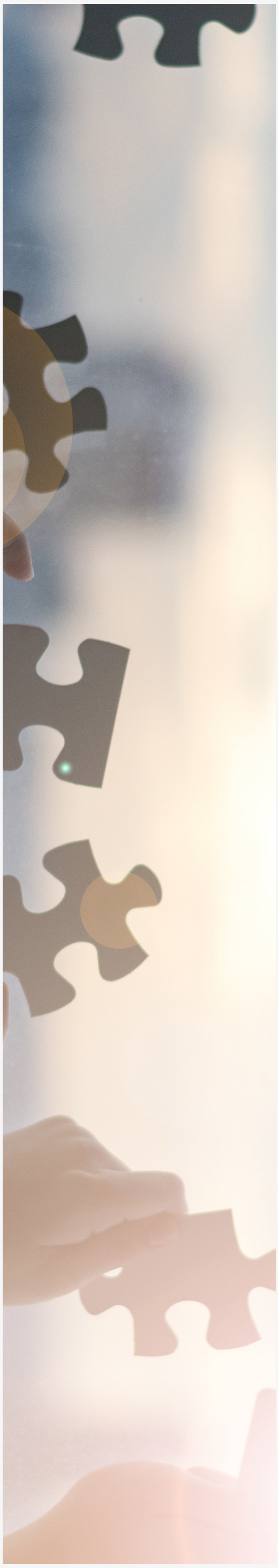

Who completed the CBPR?

For the purpose of this summary, we describe how patient-partners (youth with disabilities, parents of children with disabilities), or committee members (which could include patient-partners, researchers, and clinicians) were engaging with CHILD-BRIGHT. We also report on how well principal investigators felt they had engaged with patient-partners in their research projects. We report on the type of stakeholders presented in Table 1.

Table 1. Stakeholders who completed the CBPR survey

	2018	2019	2020
Patient-partners	40	36	18
Committee members	5	7	11
Researchers	22	11	12
Other/Not specified	39/61	35/3	20/1
TOTAL	167	92	62

Note. "Other" = staff, trainees, and co-investigators

What did we find using the CBPR?

- In **2018**, at the start of CHILD-BRIGHT projects, 86% of researchers felt that they engaged with patient-partners in their CHILD-BRIGHT research to a moderate or great extent. Most patient-partners agreed that they felt comfortable sharing their opinions with CHILD-BRIGHT (91%), that it was a true partnership (83%), that they were satisfied with their level of involvement (84%), and that there were high levels of trust (88%). The highest levels of involvement occurred in areas of developing research questions or deciding on issues to research.

- In **2019 and 2020**, when projects were underway, all researchers felt that they had engaged to a great degree with patient-partners. In 2019, all patient-partners reported agreeing to feeling comfortable sharing their opinions. For instance, 81% agreed that their engagement represented a true partnership, and 89% noted that they were satisfied with their level of involvement. In 2020, all patient-partners had high levels of trust and noted being satisfied with their level of involvement, with the highest level of patient-partner involvement occurring in the areas of creating research instruments or guidelines, and in disseminating or sharing findings.

- Looking across time points, responses were consistently very positive. The pattern showed growing patient engagement and a deepening of the quality of the partnerships.

"Even though the research questions and ideas were set four years ago, patients still feel like they are providing input into the project as it evolves. And projects are making changes based on that patient input!

The fact that every single patient-partner surveyed in 2019 felt comfortable sharing their opinion goes a long way to showing the trust that has been built in the CHILD-BRIGHT projects." Carrie Costello, CHILD-BRIGHT Parent Peer Mentor

How has patient engagement measurement unfolded since then?

In 2020, we aimed to further enrich our understanding of patient engagement and proceeded to conduct project-specific engagement evaluations. We introduced a new evaluation approach, using an instrument called the Public and Patient Engagement Evaluation Tool (PPEET).[2]

What is the PPEET and how was it administered?

The PPEET is a standardized survey that evaluates patient engagement processes and impacts. It includes questions covering four distinct areas:

- Communication and Supports for Participation;
- Sharing Views and Perspectives;
- Impacts and Influence of Engagement Initiative; and
- Final Thoughts/Satisfaction.

Each area also includes an open-ended question to allow respondents to further describe their perspectives. To align with each project within the network, questions were modified to be project specific. Similarly to the CPBR, the PPEET was administered online.

Who completed the PPEET?

A total of 105 Network members completed the PPEET surveys, including:

- Researchers: 44 individuals across 12 research projects
- Committee members who were not patient-partners: 31 individuals across 6 committees
- Committee members who were patient-partners: 8 individuals across 4 committees
- Patient-partners on research projects: 18 individuals across 8 projects
- National Youth advisors: 4 individuals.

What did we find using the PPEET survey?

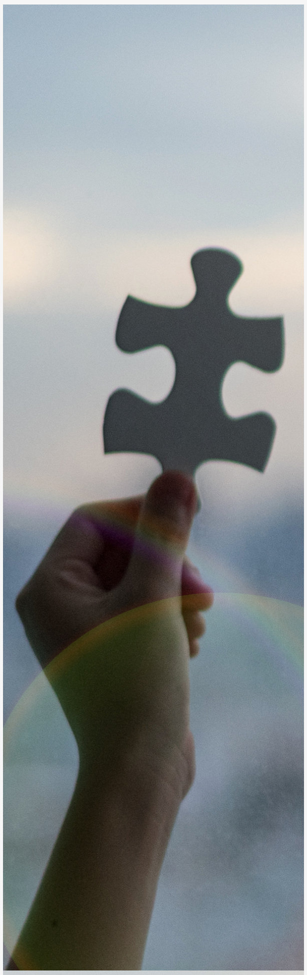

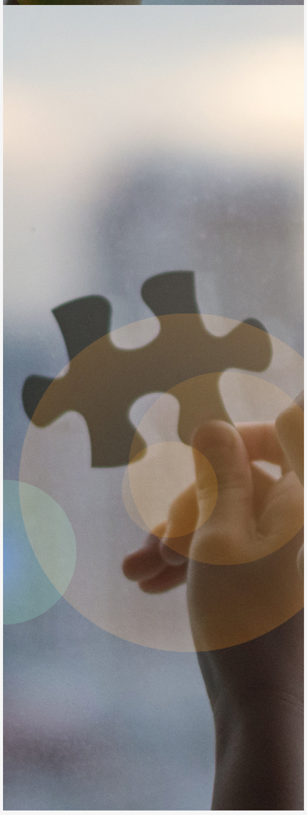

In all four areas of the PPEET, researchers' responses ranged between 'Agree' to 'Strongly agree' 87-100% of the time depending on the question. On average, 93% of researchers responded "Agree" or "Strongly agree" to the questions being posed. For example, researchers "agreed" or "strongly agreed" that patient-partners have the supports they need to participate and can share their views freely. In addition, they are also confident that the input provided by patient-partners is making a difference in the work of research projects.

Similarly, committee members' responses ranged between 'Agree' and 'Strongly Agree' from 75-96% of the time (88% on average). For patient-partners, the responses ranged between 'Agree' to 'Strongly Agree' 67-100% for all four sections (91% on average), with youth having 100% of responses 'Agree' or higher for every question on the PPEET.

Overall, patient engagement and its impacts were consistently highly rated across the different CHILD-BRIGHT Network stakeholder groups, especially within research project teams. Researchers, committee members, and patient-partners reported high levels of satisfaction with patient engagement in the Network.

A preliminary overview of the responses to the open-ended questions of the PPEET both highlighted areas where activities have gone particularly well and identified areas needing improvement. Patient-partners noted that early involvement helped enable greater impact on the research project and its advancement, and that regular and detailed communication kept them engaged. Several said that their engagement had deepened over time. On the other hand, greater diversity (e.g. more fathers and people from a greater range of ethnic and racial backgrounds) is needed. Furthermore, in some instances, greater role clarity for patient-partners would be beneficial.

Figure 1. Agreement to PPEET questions

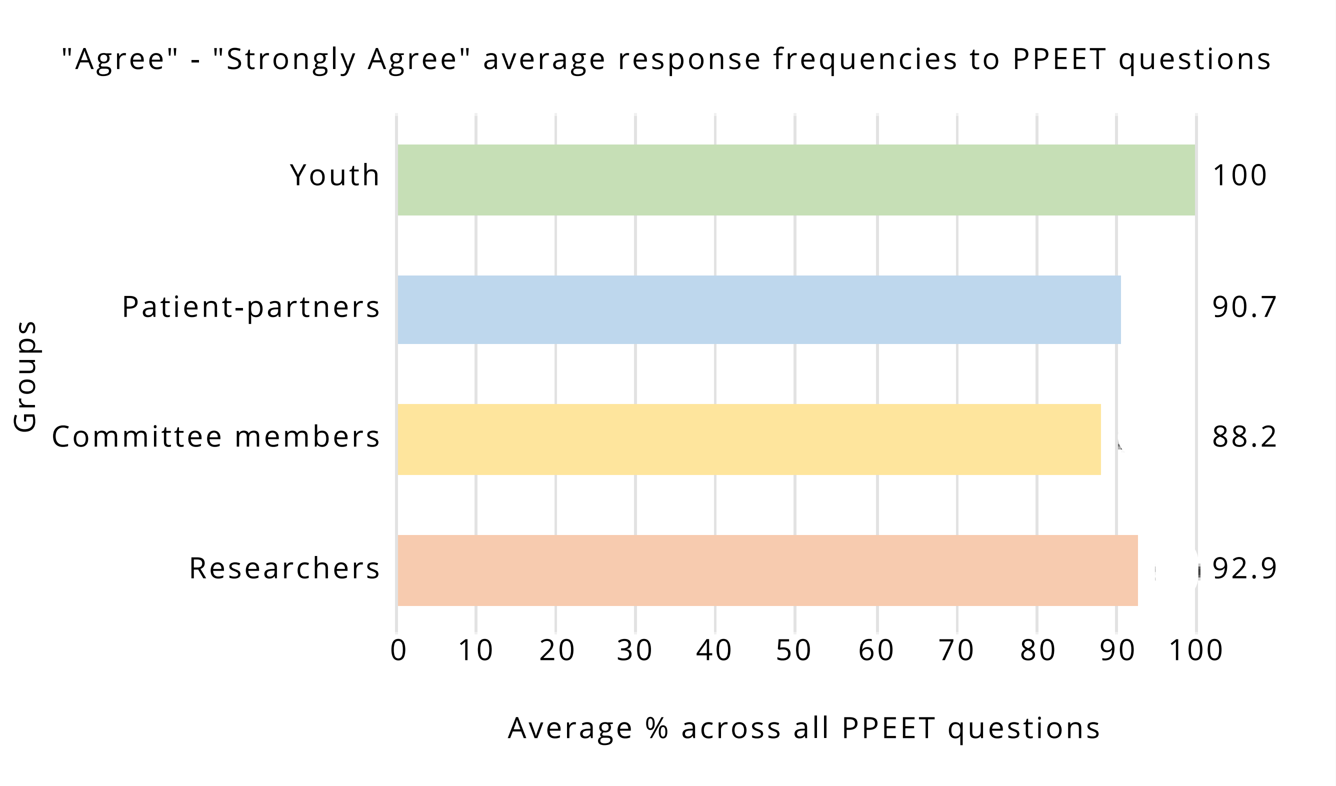

Example of a PPEET question: Patient-partners have the supports they need to participate (e.g. meetings time and methods, travel, accommodations, etc.).

What is the take home message?

Our findings to date suggest that the CHILD-BRIGHT Network is effectively implementing strategies for authentic and impactful patient-oriented research. Overall, patient-partners are satisfied with their level of engagement in the Network's research and governance. The quality of patient engagement is highly rated across the different stakeholder groups of the CHILD-BRIGHT Network.

The CHILD-BRIGHT Network has 13 different research projects. In refining our evaluation approaches over time, we determined that patient engagement was positively rated within the different projects. These results are encouraging given that we partner with members across a large, nation-wide network, where connections are primarily virtual, and most members were novices to the patient-oriented research process at the onset of the Network's activities.

WE AUTHENTICALLY PARTNER WITH MEMBERS ACROSS A LARGE, NATION-WIDE NETWORK.

Moving forward, what are our next steps?

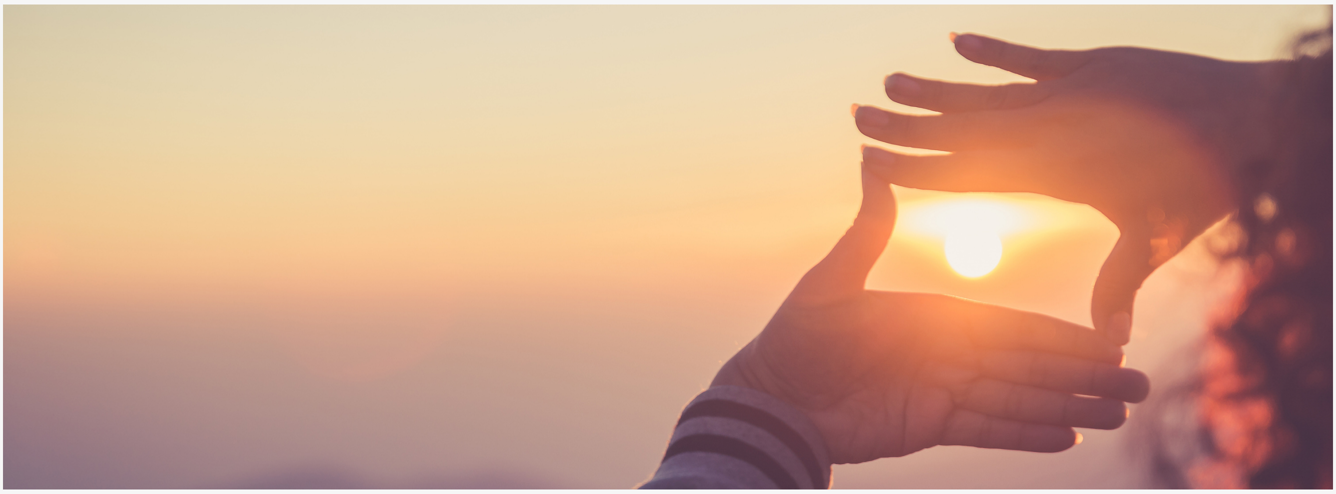

Our evaluations have also shown us **areas for improvement**. Some respondents reported challenges around "balancing [their] availability to the requests made." Others mentioned needing more effective communication to clarify project goals and to stay actively involved; as well as needing to increase the diversity of our group members.

The open-ended responses to the CBPR and PPEET surveys are to be analyzed in more detail in the near future. To enrich our understanding of the patient engagement experience, the Network has also conducted semi-structured interviews with different stakeholders to complement those findings, which are currently being analyzed.

Read what Lena Faust, CHILD-BRIGHT Youth Member, has to say about:

Enhancing engagement/involvement:
"I would say that working towards more early-stage engagement with stakeholders in all areas of the research process, from identifying priorities to developing questions, could be an area of focus to enhance engagement."

Network cohesion, clarity of goals/outputs:
"I think defining clear goals and specific outputs for stakeholder involvements would be very helpful. Clear messaging and goals are key!"

The Report

The CHILD-BRIGHT Network Measuring Patient Engagement Working Group. Stakeholder Engagement in the CHILD-BRIGHT Network, 2018 to 2020, Preliminary Findings [Internet]. October 2021.

Acknowledgement

This report was prepared by the CHILD-BRIGHT Network Measuring Patient Engagement Working Group in collaboration with a CHILD-BRIGHT parent-partner and a youth.

References

1. The University of New Mexico, Health Sciences Center, Center for Participatory Research. CBPR Project: Research for Improved Health 2009 – 2013 [Internet]. Available from: https://cpr.unm.edu/research-projects/cbpr-project/research-for-improved-health.html

2. McMaster University Faculty of Health Sciences. PPEET Public and Patient Engagement Evaluation Tool [Internet]. Available from: https://ppe.mcmaster.ca/our-products/public-patient-engagement-evaluation-tool

www.child-bright.ca

CHILD-BRIGHT Network
5252 Boul. de Maisonneuve Ouest
Montréal (Québec)
H4A 3S5
admin@child-bright.ca
