# Supplementary material for: Patient engagement in a national research network: barriers, facilitators, and impacts
Source: Res Involv Engagem. 2023 Mar 8;9:7. doi: 10.1186/s40900-023-00418-5 (PMC9993369; doi:10.1186/s40900-023-00418-5)
Supplement: Supplementary file 2 — Additional file 2. “Patient engagement in CHILD-BRIGHT’s patient-oriented research Network: Scratching beneath the surface” contains the April 2022 research brief produced by CHILD-BRIGHT. [file 40900_2023_418_MOESM2_ESM.pdf]

CHILD-BRIGHT Network

Strategy for Patient-Oriented Research SPOR Putting Patients First

RESEARCH BRIEF

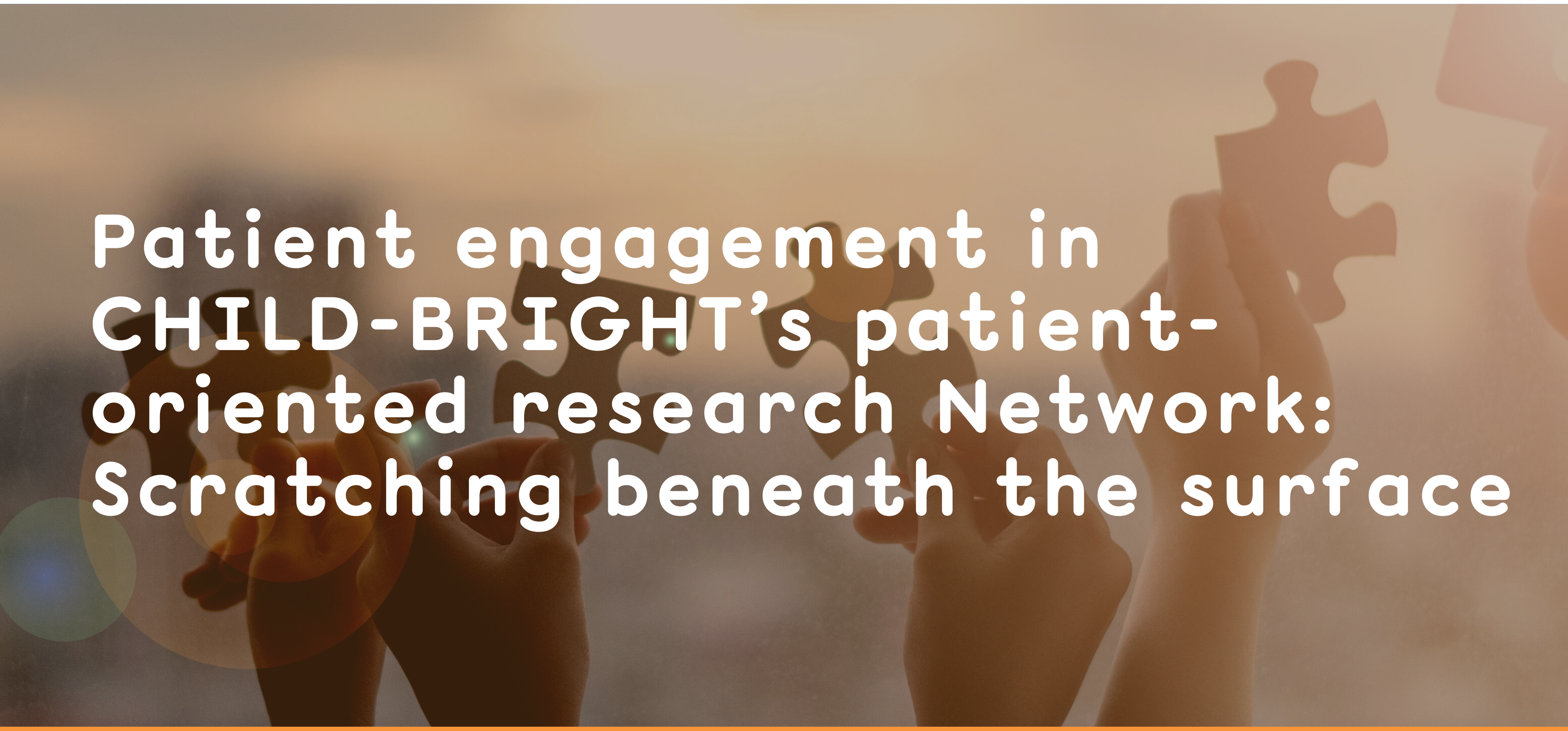

Patient engagement in CHILD-BRIGHT's patient-oriented research Network: Scratching beneath the surface

Stakeholder Engagement in the CHILD-BRIGHT Network, Part 3 April 2022

What is this research brief about?

In this brief, we report on the results of qualitative interviews conducted to gain a better understanding of the engagement experiences of CHILD-BRIGHT Network members. In this brief, you will find the following information:

- **Section 1**: What is the current situation? (page 2)
- **Section 2**: What did CHILD-BRIGHT do? (page 2)
- **Section 3**: What were the main interview findings? (page 3)
  Four sub-themes emerged from the data and are briefly presented:
  - 3.1. Barriers and facilitators to engagement with research projects (page 3)
  - 3.2. Barriers and facilitators to wider Network engagement (page 8)
  - 3.3. Impacts of patient-oriented research (page 10)
  - 3.4. Solutions to improve engagement (page 12)
- **Section 4**: What should CHILD-BRIGHT prioritize as next steps? (page 13)

The study was conducted in collaboration with various CHILD-BRIGHT stakeholders including patient-partners, the Knowledge Translation committee, and the Citizen Engagement Council.

1. What is the current situation?

Given CHILD-BRIGHT's commitment to meaningfully engaging patient-partners and improving engagement levels, evaluating Network member engagement and its impact is an ongoing activity. Since 2018, we have been using standardized instruments such as the Community-Based Participatory Research (CBPR) questionnaire, and more recently, the Public and Patient Engagement Evaluation Tool or PPEET (see our report highlighting preliminary findings here). Data collected through these instruments suggest high levels of engagement among patient-partners. An in depth understanding of what facilitates or impedes this level of engagement was missing as was an understanding of impacts of patient-oriented research at CHILD-BRIGHT.

2. What did CHILD-BRIGHT do?

To better interpret the results of the standardized instruments we have been using (e.g., CBPR, PPEET) and to capture elements not included in these tools, we decided to add a qualitative component, i.e., interviews, to our patient engagement measurement approach.

Purpose of the interviews

The purpose was to improve our understanding of the barriers and facilitators of patient engagement and the impacts of patient-oriented research from patient-partners' and researchers' perspectives.

Data collection

All CHILD-BRIGHT Network members were invited to participate in a 30-minute interview. Consent was obtained prior to each interview. The interview guide centered around three main topics: (1) the interviewees' involvement at CHILD-BRIGHT, (2) the benefits, challenges, and supports to engagement, and (3) interviewees' perceived impact of patient-oriented research. We conducted interviews in English between summer and fall of 2020 via Zoom (a video conference platform) and obtained Ethics approval from McGill University Health Centre's Research Ethics Board.

Data analysis

Two researchers trained in qualitative data analysis used a content analysis approach to analyze the data (identification and creation of codes, categories, and themes). The data coded by one researcher was reviewed for consistency of coding by the other researcher and disagreements were resolved through discussion.

Stakeholder involvement

Two parents of children with brain-based developmental disabilities were patient-partners on this project: one worked with us on all phases of the project and the other was an interviewer on the team. We also asked CHILD-BRIGHT's Citizen Engagement Council and Knowledge Translation Committee to review this research brief and provide feedback regarding: (1) language, (2) clarity, and (3) next steps CHILD-BRIGHT should prioritize given the findings.

Interview participants

A total of 25 Network members participated in the interviews: 7 male and 18 female. In terms of stakeholder group group, 12 were patient-partners and 13 were researchers.

3. What were the main interview findings?

One overarching theme with four sub-themes emerged from the data (Figure 1).

3.1. Barriers and facilitators to engagement with research projects

3.1.a. Barriers

Interviewees spoke about barriers to engagement with projects. The tables below list the top five barriers commonly reported by patient-partners and researchers.

The interview quotes below have been edited lightly for clarity.

A total of 25 Network members participated in the interviews. Interviewees spoke about the barriers and facilitators to engagement.

Figure 1. CHILD-BRIGHT Network members' engagement experience: overarching theme and sub-themes

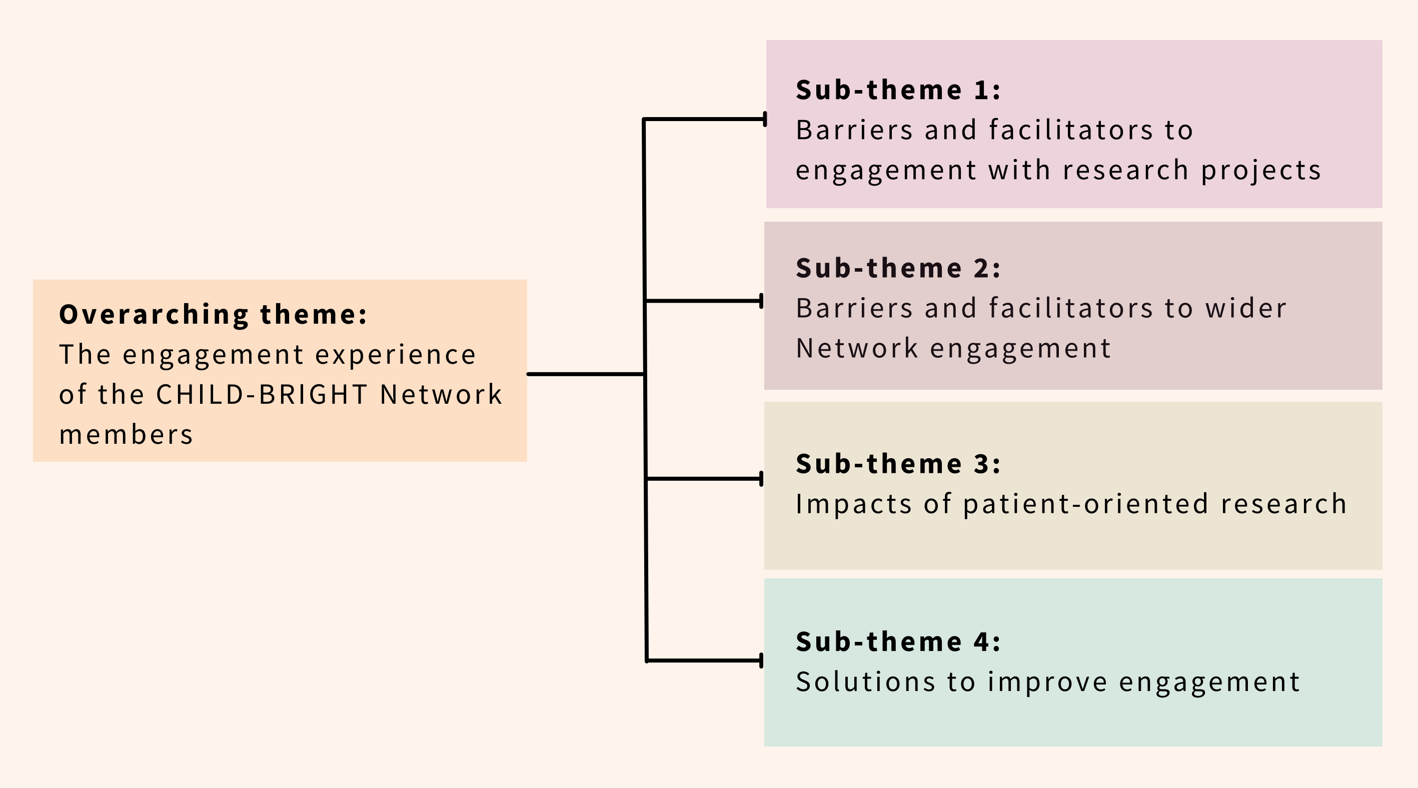

Table 1. Barriers to **engagement with research projects** most frequently reported by **patient-partners**

Categories	Sample Quote	Utterances n/57*
**Communication challenges**<br>Unclear expectations and roles; Lack of follow-up; Logistics of meetings; Being in different time zones; Lack of in-person communication; Feeling excluded; Use of academic jargon.	"Sometimes communication can be a challenge in terms of assumptions about what I would think my role would be. They would have a different idea than what I would."	25
**Factors specific to patient-partners**<br>Time limitation and working schedule; Lack of related experience; Role recognition; Homogeneity of patient-partners; Engagement can be too scientific and methodological for patient-partners.	"When I started out, I had no idea of how much time [I'd spend]. I'm spending much more time than I expected to spend on it… this varies a lot but certainly in at least two full days a week on average."	13
**Difficulty maintaining engagement over time**	"Waiting, having a patient-partner sit on the sidelines doing nothing for four or five months while [Research Ethics Board] approval is gained, might seem like nothing to the research team, but it may be a whole lot to a particular patient-partner who says, 'I'm out of the loop completely'. They might find it very hard to get re-engaged."	6
**Learning to work together and achieve genuine collaboration**	There's been a challenge sometimes in achieving genuine collaboration. Sometimes that means being candid with people. […] . What happens sometimes is there's a dynamic whereby you're there to point out problems and to press for change."	4
**Lack of patient-partner feedback integration**	"Not everything I say is relevant, or should be taken into account but I know the researchers on [a project external to CHILD-BRIGHT] are very, very careful, even if they're going to dismiss what we say, they are very careful as to tell us why… that is not the case with every single researcher that I've come in contact with at CHILD-BRIGHT."	4

Note. 57* refers to the total number of instances patient-partners spoke about barriers to engagement with projects.

Table 2. Barriers to **engagement with research projects** most frequently reported by **researchers**

Categories	Sample Quote	Utterances n /42*
**Communication challenges** Unclear expectations and roles; Lack of follow-up; Logistics of meetings; Being in different time zones; Lack of in-person communication; Lengthy questionnaires; Representativeness of advocates.	"That was another big piece, I think, for [patient-partners]. We kept hearing from them, like we, 'At some point even if you can't do it right now, we would like to see, and hear, and understand, a summary of some of the things we talked about, and the evolution of those ideas, if they were taken up or if they weren't, why they weren't.' I think closing the loop has been a real big challenge for us. Even though we know it's important, and we value it, it's an extra step right that we don't typically do when we're a research project. We just make decisions, and we move forward with them."	11
**Lack of guidelines, framework, and structure** Lack of guidelines about how and when to engage; Recruiting patient-partners; Infrastructural support; Engagement curriculum/framework.	"Very quickly, I realized that these families, we needed a formal curriculum for [patient engagement]. For these families, this is not something that comes naturally. Just engaging the family because we like to hear their opinion is not what is needed here. There is a skill set that the rest of us had that these families did not have, as gifted as they were, to do this. So, I feel like there are certain parts of family engagement that require curriculum and training that we did not have."	7
**Learning to work together and achieve genuine collaboration**	"A big thing that we found early on, with a little bit of pushback maybe with some of the more seasoned parents that were part of the group, was that we need to work a little bit to make [the partnership] reciprocal. So that it felt like we weren't always only talking about what we need ... and that we were listening to them."	5
**Difficulty maintaining engagement over time**	"… You have to think about how and when to engage patient-partners to value their time. Sometimes research can be very slow and it's a bit discouraging sometimes for patient- and family partners to be involved because they're all excited to be a part of it, but then research projects go on for years and years […] and things haven't really evolved, and so I think sometimes that can be a bit disheartening."	4
**Factors specific to patient-partners** Time limitation and working schedule; Lack of related experience; Role recognition; Homogeneity of patient-partners.	"[Our patient-partners are] dealing with people who never know whether their kid is going to be in hospital tomorrow, [be] sick or ha[ve] a seizure. The complexity of their lives makes it extraordinarily difficult to ask them to commit to something… so it requires a lot more flexibility."	4

Note. *42 refers to the total number of instances researchers spoke about barriers to engagement with projects.

3.1.b. Facilitators

Patient-partners and researchers also spoke about what helped support or facilitate their engagement with research projects. Tables 3 and 4 list facilitators commonly reported by patient-partners and researchers, respectively.

Table 3. Facilitators to **engagement with research projects** most frequently reported by **patient-partners**

Categories	Sample Quote	Utterances n/65*
**Communication** Having national/group meetings; Using different methods of communication; Seeing CHILD-BRIGHT as a safe space to speak up; Open communication; Face-to-face interactions; Check-ins.	Upon learning that there is a Zoom phone app, one patient-partner shared: "I can [now] walk around with my earbuds and still administer medications or check on my son [during meetings]. So, that was wonderful to know that those resources were available."	29
**Patient-partner qualities** Experience/skills of patient-partners; Motivation and commitment.	"We all come from different backgrounds. Many of us have several degrees, many of us have our own businesses, or jobs that we do in addition to parenting and that can actually be useful too in the work that [researchers] do."	8
**Respect, trust, and partnership** Importance of mutual respect and trust between patient-partners and researchers; importance of reciprocal partnerships where both parties benefit.	"The initial engagement [was really good]. I think it was a two-day meeting at McGill and there were a lot of brainstorming sessions. When you take the time and effort to do that face to face, and everybody gets to know one another and knows what they're there for, why they're there and what they hope to get out out of it, you do have that kind of respect and trust that then informs the rest of the engagement [...] If you don't do that, there's no sense of trust so you don't share as much, and you may not speak out as much."	5
**Researcher-specific qualities** Openness of researchers to feedback and collaboration; Previous connection to researchers.	"Sometimes it comes down to really simplistic things. For one project I was with previously, we all got together at a research conference. It was nice when they said, 'We want you to present the poster'. I thought that was just really wonderful in the sense of saying, 'Well, you're a part of the team, you can explain as well.'"	5
**Integrating feedback and explaining why feedback can't be used**	"They validated what we talked about, they didn't gloss it over like a professional can do or simplify it. I mean, I'm a better professional for working with them, to be honest, because they listen, they also model how to integrate different perspectives."	4

Note. *65 refers to the total number of instances patient-partners spoke about facilitators to engagement with projects.

Table 4. Facilitators to **engagement with research projects** most frequently reported by **researchers**

Categories	Sample Quote	Utterances n/69*
**Communication**<br>Having national/group meetings; Using different methods of communication; Seeing CHILD-BRIGHT as a safe space to speak up; Open communication; Check-ins.	"The way the conferences have been run has been quite a success in terms of bringing a lot of patient family partners together in the conference along with researchers [...] I think that's been a real strength and it's quite visible compared to other research areas. And I think it gives people a platform where they are elevated to equal partners rather than observers."	14
**Patient-partner qualities**<br>Experience/skills; Motivation and commitment; Bringing different perspectives to the table.	"Sharing perspectives has been [very useful] for our particular group, where there's people from a variety of backgrounds. That is very, very useful, because the more perspectives you can bring in from people from different backgrounds that are connected somehow, or stakeholders, I think that can only improve the direction."	6
**Flexibility**<br>CHILD-BRIGHT providing flexibility to researchers in participation, extensions, scheduling meetings.	"For our project we've had to extend our timeline a few times and CHILD-BRIGHT hasn't put up any barriers in doing that. In fact, they've been very supportive knowing that to do work at the level of engagement that they want and that we want, it was a no-brainer to extend the timeline. [They even] provid[ed] us a little bit more funding to do that."	6
**Respect, trust, and partnership**	"Entering into these partnerships with respect, it is very important."<br><br>"There's reciprocity built-in knowing that we will gain from [these partnerships]—whether it's our careers, whether it's our institutions—our networks will have monumental benefits from collecting data with [...] communities and we need to be cognizant that there needs to be a high level of reciprocity in the community."	5
**Compensation and flexibility in compensation**	"It's increasingly recognized that you should be paying people who are part of the team. [. . .] but there's also the feeling that you don't want to coerce people into participating by offering them money that they become dependent on. And the third thing is that it can actually become bureaucratically very cumbersome. You know, if people are having to file taxes for this, you have to make it worth their while to even go through the paperwork."	5

Note. *69 refers to the total number of instances researchers spoke about facilitators to engagement with projects.

3.2. Barriers and facilitators to wider Network engagement

Interviewees spoke about wider Network engagement. The tables below highlight barriers and facilitators commonly reported by patient-partners and researchers.

3.2.a. Barriers

Table 5. Barriers to wider Network engagement reported by **patient-partners**

Categories	Sample Quote	Utterances n/38**
**Communication challenges**<br>Lack of information about CHILD-BRIGHT Network activities/initiatives, Lack of information about research projects; Lack of plain language when communicating; Network communication issue*; Too many emails.	"Language is a roadblock. It's up to people like me to go and remind everybody [to use plain language]."	13
**Factors specific to patient-partners**<br>Limited time; Mismatch between the patient-partner's experience and views and the research focus; Patient-partner shyness.	"I guess time commitments... I also have a full-time job."<br><br>"A lot of the very pediatric-focused questions that CHILD-BRIGHT is asking, I kind of feel like I'm not necessarily the best person to answer those questions a lot of the time."	8
**Time commitment required and asking too much of patient-partners**	"The time commitment, it's, it's huge, you know, […] It's just a huge commitment. I'm considering in the next round, you know, whether I stay or just, I don't know. I love it, but I have to figure out if I can do it."	7
**Power imbalances**<br>Patient-partners not seen as equal to researchers.	"It is an interesting power dynamic if someone has more power […] and in some ways because the researchers have all the money, they have all the power. […] So, there is that power dynamic, regardless of these labels with experts and things."	6

Notes.
*Network communication issue refers to how to ensure information reaches everyone (executive team, patient-partners, etc.).
**38 refers to the total number of instances patient-partners spoke about barriers to wider Network engagement.

Table 6. Barriers to wider **Network engagement** reported by **researchers**

Categories	Sample Quote	Utterances n/9*
**Time commitment required and asking too much of patient-partners**	"I've gone on the [CHILD-BRIGHT] website. I get the emails and when I have time, I'll look over stuff. But time is always an issue. "<br><br>"…But to parse our time by all of the little sub-studies that we're asked to participate in […] There's a feeling of obligation, there's a feeling of gratefulness, there's a feeling of wanting to give back and there an overwhelming feeling of guilt for not being able to keep up with all these things. "	4

Note. *9 refers to the total number of instances researchers spoke about barriers to wider Network engagement.

3.2.b. Facilitators

Table 7. Facilitators to wider **Network engagement** reported by **patient-partners**

Categories	Sample Quote	Utterances n/40*
**Factors specific to researchers**<br>Openness of researchers to feedback; Liking the researchers; Having a previous work relationship with researcher; Sense of commitment to their research.	"I think that's why I've stayed. I never felt like someone was rolling their eyes in the background at me."<br><br>"I would say that there are some really amazing researchers in CHILD-BRIGHT who are very, very supportive of patient-partners and I think that's important to say. For the most part, researchers are really careful and really caring about how they interact with patient-partners."	14
**Factors specific to patient-partners**<br>Motivation to contribute; Not being intimidated or scared.	"The benefit is we're clearing the brush away as we're forging the path and we're right at the front there. I think the intrinsic benefit is giving me that energy to keep doing that."	10
**Communication**<br>Regular follow-ups; Different methods of communication (newsletter, conferences, meeting); Accessible information (accessible format, clear, easy to ask and get questions answered).	"They're very good at, in meetings, making sure that it's in accessible format for everyone and providing translations and everything, so that's good. If we ever have any questions it's always easy to follow up [...] it's really easy to reach out… and have all your questions answered so that stuff is great."	9
**Personal connection to the project or having a role within the Network**	"I had a very strong relationship with the researchers, and I got the newsletters and things like that, but I wouldn't say I understood what the Network was or did."	5

Note. *40 refers to the total number of instances patient-partners spoke about facilitators to wider Network engagement.

Table 8. Facilitators to wider **Network engagement** reported by **researchers**

Categories	Sample Quote	Utterances n/15*
**Communication and having a supportive Network**<br>Regular follow-ups, various communication methods.	"...The fact that we've gone virtual now, that just makes it easier... for people to be part of things where maybe there were barriers before."<br><br>"In terms of support, I find the Network is very open to hearing about other ways to engage with patients and families… I think that's something that I really like about the Network, being able to reach out."	6
**Providing different engagement opportunities and establishing meaningful collaborations with patient-partners**	"I think some of them do [feel engaged], particularly because they are not just involved in one thing. So, some of our members are on other Network committees, or on other projects even. So, I think those people feel more connected to the Network, at a larger level…"<br><br>"I mean we're talking about people who have so many demands on their time, that we try very hard not to make demands that are not, that are not really important. So we don't trivialize their involvement. We structure it so that they are doing things that make a difference."	6

Note. *15 refers to the total number of instances researchers spoke about facilitators to wider Network engagement.

3.3. Impacts of patient-oriented research

Patient-partners and researchers spoke about the successes, strengths, or impacts of patient-oriented research. Below are the **top five impacts** reported by patient-partners and researchers, respectively.

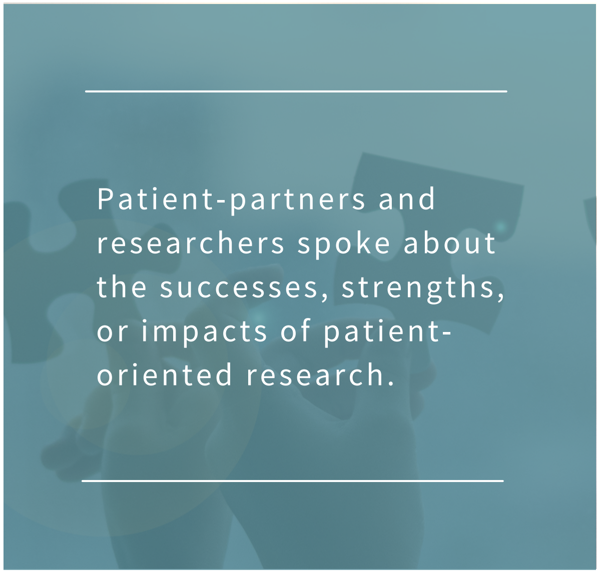

Impact reported by patient-partners (Total number of utterances: 97)	Impact reported by researchers (Total number of utterances: 100)
**1. Projects are better aligned with patient-partner priorities and lived experiences (25 utterances)**<br><br>“This particular project has been really lovely in the way that they have really taken all of our input and our perspectives, because the end user is folks like us.”	**1. Projects are better aligned with patient-partner priorities and lived experiences (32 utterances)**<br><br>“It ensures that the research is practically oriented and applied, and the language used is simple. There are often family members or self-advocates that are really pushing for the research to be used, and relevant and asking pertinent questions. So I think it does change the tone, for sure.”
**2. Patient-partners are provided with learning opportunities and experiences (22 utterances)**<br><br>“And I think sometimes it comes down to really simplistic things. With one project I was with previously, we all got together at a research conference. It was nice they said, ‘we want you to present the poster’. And I thought that was just really, wonderful in the sense of saying, ‘well you’re a part of the team, you can explain as well.”	**2. Co-creation and collaboration among researchers, patient-partners, and families increases (27 utterances)**<br><br>“Having the patient-partners very much involved makes any strategic decisions more realistic, more impactful. So, I think for strategic decisions, having patient-partners is really important to make sure that the patient experience and patient expertise is really influencing our decision making.”
**3. Co-creation and collaboration among researchers, patient-partners, and families increases (18 utterances)**<br><br>“I think that a lot of how I frame things [as a parent-partner], or how I look at things has had a really big impact in how we're reporting, how we're measuring, how we're designing the program…”	**3. Knowledge translation is facilitated by patient-partner input (11 utterances)**<br><br>“The incredible students that we’ve had have been able to listen to the parents’ feedback, take very specific feedback and turn it into resources for families.”
**4. Knowledge translation is facilitated by patient-partner input (10 utterances)**<br><br>“ We're redesigning a program that was made for a certain cohort of the disabled population and now we're rejigging it so the videos have to change, some of the language has to change […] because it’s for different disabilities, for children or for parents of children with different kind of disability.”	**4. Members can learn about patient-oriented research together (6 utterances)**<br><br>“I think we try to be open as much as possible and did see it as a learning process together and I think that was appreciated that we were open in that regards.”
**5. Members can meet others, share experiences, increase knowledge and know they are not alone (8 utterances)**<br><br>“I would count several of the parent advisors as friends now, or certainly colleagues that I can reach out to on issues not related to the study. So yes, it has enhanced my knowledge of childhood disability… I have been able to interact with parents with kids who have different kinds of disabilities or comorbidities and also different challenges…. I’ve had the chance to meet with some parents as well, which I’m very happy about.”	**5. The voices of patient-partners are amplified (5 utterances)**<br><br>“By having these parents who are willing—they're very busy but, they're willing because they think it's important to have their voices heard and to speak on behalf of other parents—I think that having that opportunity is really helpful for the study.”

3.4. Solutions to improve engagement

Finally, both patient-partners and researchers shared ideas for improving engagement at CHILD-BRIGHT. Solutions commonly reported by patient-partners and researchers are highlighted below.

**Patient-partners**<br>Total number of utterances: 36	**Researchers**<br>Total number of utterances: 44
**Adapt (13 utterances)**<br>Adapt communication within projects depending on target audience, time zones, expected length of project.	**Adapt (6 utterances)**<br>Adapt communication within projects depending on target audience, time zones, expected length of project.
**Diversify (8 utterances)**<br>Diversify by engaging different voices.	**Diversify (5 utterances)**<br>Diversify by engaging different voices.
**Follow-up (5 utterances)**<br>Follow-up and check-in with patient-partners and support new patient-partners.	**Follow-up (5 utterances)**<br>Follow-up/check-in with patient-partners and support new patient-partners.
**Match (4 utterances)**<br>Match patient-partner interest with Network needs.	**Match (5 utterances)**<br>Match patient-partner interest with Network needs.
	**Improve (5 utterances)**<br>Improve Network communication such as emails and newsletters to better showcase initiatives, accomplishments, stories.
	**Evaluate (5 utterances)**<br>Evaluate engagement and compensation models.
	**Learn (4 utterances)**<br>Learn about patient-oriented research/ how to engage.

Notes.

- **"Adapt communication"** refers to adapting communication within the research project depending on the target audience (such as using lay language), keeping time zones in mind for conference calls, face-to-face interactions, and email, clear communication about the expected length of a project and timelines of follow-up.
- **"Follow-up/Check-in with patient-partners and support new patient-partners"** refers to keeping patient-partners updated on project progress (providing information) and supporting new patient-partners by providing information on expectations and objectives and offering training.

4. What should CHILD-BRIGHT prioritize as next steps?

CHILD-BRIGHT's Citizen Engagement Council (CEC) and Knowledge Translation Committee were asked to review this brief and provide feedback regarding language, clarity, and next steps that should be prioritized by CHILD-BRIGHT given the findings. Comments were reviewed by two research team members and recommendations are listed below.

Identify strategies to improve engagement. This would involve identifying:

1. Steps to actualize the proposed solutions (e.g., how to adapt communication within projects, how to diversify, etc.),
2. Who would be responsible for developing the action plan and leading the various parts, and
3. When and how to implement the action plan.

Specific recommendations to develop the identified solutions were also provided:

- Re-engage patient-partners
- Improve communication from projects and committees to patient-partners (sending out meeting and time zone reminders, for example).
  - Email updates or check-ins between meetings that relate to the Connections newsletter which is dedicated to CHILD-BRIGHT members. Select content from the newsletter that would be interesting for patient-partners or that is related to the work they are involved in.
  - Yearly reminders from Citizen Engagement to project teams to support and encourage them in communicating with patient-partners.
- Conduct focus groups with patient-partners and researchers to discuss engagement
- Develop relationships in less formal ways among members (e.g., informal get-togethers, Facebook group).

Specific recommendations:

- Re-engage patient-partners
- Improve communication
- Conduct focus groups to discuss engagement
- Develop relationships in less formal ways

Acknowledgements

We would like to thank:

- CHILD-BRIGHT Network members who took the time to share their experiences with us and participated in the interviews
- CHILD-BRIGHT's Citizen Engagement Council and Knowledge Translation Committee for reviewing this brief and providing suggestions for next steps
- Amy Spurway, CHILD-BRIGHT Network patient-partner and interviewer
- Jacqueline Di Bartolomeo, CHILD-BRIGHT Network staff and interviewer
- Olivia Vadacchino, research assistant on this project.

Suggested citation:
Gonzalez, M., Ogourtsova, T., Zerbo, A., Lalonde, C., Spurway, A., Gavin, F., Weiss, J., Shikako, K., & Majnemer, A. (April 2022). Patient engagement in CHILD-BRIGHT's patient-oriented research Network: Scratching beneath the surface. Research brief submitted to CHILD-BRIGHT's Measuring Patient Engagement Working Group.

Brief design by: Alix Zerbo, MPA, MSc.

Research team:
Evaluating patient engagement and its impact is an ongoing activity at the CHILD-BRIGHT Network. Research activities for this purpose including interview work reported in this brief have been overseen by **CHILD-BRIGHT's Measuring Patient Engagement Working Group:**

- Frank Gavin, Former Director, Citizen Engagement Program, Former Chairperson, Citizen Engagement Council, CHILD-BRIGHT Network
- Miriam Gonzalez, MSc., PhD, Postdoctoral Fellow, Faculty of Medicine, McGill University
- Corinne Lalonde, M.A., Citizen Engagement Program Coordinator/Project Manager, CHILD-BRIGHT Network
- Alix Zerbo, MPA, MSc., Knowledge Translation Coordinator/Information Officer, CHILD-BRIGHT Network
- Jonathan Weiss, PhD, Knowledge Translation Program Co-Lead, CHILD-BRIGHT Network, Professor, Faculty of Health, York University
- Annette Majnemer, PhD, Director, CHILD-BRIGHT Network, Faculty of Medicine and Health Sciences, McGill University

CHILD-BRIGHT Network | Strategy for Patient-Oriented Research SPOR Putting Patients First

CHILD-BRIGHT Network
5252 Boul. de Maisonneuve Ouest
Montréal (Québec) H4A 3S5
admin@child-bright.ca
www.child-bright.ca
