# Supplementary material for: Patient engagement in a national research network: barriers, facilitators, and impacts
Source: Res Involv Engagem. 2023 Mar 8;9:7. doi: 10.1186/s40900-023-00418-5 (PMC9993369; doi:10.1186/s40900-023-00418-5)
Supplement: Supplementary file 3 — Additional file 3. “GRIPP2 PPI reporting Checklist” contains the results of the GRIPP2 short form reporting checklist. [file 40900_2023_418_MOESM3_ESM.pdf]

Additional File 3

GRIPP2-SF PPI reporting Checklist

Section and topics	Item	Reported on page No
1: Aim	Report the aim of PPI in the study	5-6
2: Methods	Provide a clear description of the methods used for PPI in the study	6-8
3: Study results	Outcomes—Report the results of PPI in the study, including both positive and negative outcomes	9-17
4: Discussion and conclusions	Outcomes—Comment on the extent to which PPI influenced the study overall. Describe positive and negative effects	17-21
5: Reflections/critical perspective	Comment critically on the study, reflecting on the things that went well and those that did not, so others can learn from this experience	20-21

PPI patient and public involvement
