# Supplementary material for: Patient engagement in a national research network: barriers, facilitators, and impacts
Source: Res Involv Engagem. 2023 Mar 8;9:7. doi: 10.1186/s40900-023-00418-5 (PMC9993369; doi:10.1186/s40900-023-00418-5)
Supplement: Supplementary file 4 — Additional file 4. “Interview Guide Questions” contains the qualitative interview questions used for patient-partners and researchers. [file 40900_2023_418_MOESM4_ESM.pdf]

Additional File 4

Interview Guide Questions

- Qualitative Interview questions for patient-partners

1. Benefits:
   How do you feel you've benefited from your involvement in ____specific phrase________?
   How do you feel the researchers have benefitted from your involvement?

2. Challenges:
   What challenges, if any, did you experience as a research partner or committee member?
   What helped ease or overcome these challenges?

3. Supports:
   What helped to support your engagement with your research project or committee work?
   Did any particular person or action help make your engagement easier?
   What other supports or actions would have helped make engaging or contributing easier?
   A) Did you have contact with the parent peer mentor?
   B) What was your experience with the parent peer mentor like?

4. Network engagement:
   Do you feel engaged with or connected to CHILD-BRIGHT as a network? Please describe why or why not.

5. Impact:
   In your experience with CHILD-BRIGHT, have you noticed anything that demonstrates the successes or the strengths of patient-oriented research? Please describe.

   What impact, if any, do you feel you've had on changing or shaping the research project?

   What impact, if any, do you feel you've had on changing or shaping the work of the committee?

   What impact, if any, do you feel you've had on changing or shaping the direction of CHILD-BRIGHT?

   What impact, if any, do you feel your work with CHILD-BRIGHT has had on the futures of children with disabilities and their families?

- Qualitative Interview questions for researchers

1. Benefits: Do you think the research benefited from patient-partner involvement? If so, what were those benefits?

2. Challenges: What did you find challenging about collaborating with patient-partners in a research team, if there was anything challenging? What helped you overcome these challenges?

3. Supports: What did you find helpful in your efforts to incorporate patient-partner perspectives and input into the research project? Can you describe a particular strategy that you used to promote patient-partner engagement over the past years? What additional supports would have helped you in your effort to engage patient-partners?

4. Network engagement: Do you believe the patient-partners of your research project feel engaged with or connected to CHILD-BRIGHT as a Network? If so, in what ways?

5. Impact: Have you taken note of any successes of patient-oriented research as a way of doing research with CHILD-BRIGHT? If so, what were those successes? What impact, if any, do you believe patient-partner engagement had in changing the research project?
